# The importance of nerve microenvironment for schwannoma development

**DOI:** 10.1007/s00401-016-1583-8

**Published:** 2016-05-28

**Authors:** Alexander Schulz, Robert Büttner, Christian Hagel, Stephan L. Baader, Lan Kluwe, Johannes Salamon, Victor-Felix Mautner, Thomas Mindos, David B. Parkinson, Jeffrey R. Gehlhausen, D. Wade Clapp, Helen Morrison

**Affiliations:** Leibniz Institute on Aging, Fritz Lipmann Institute, Beutenbergstrasse 11, 07745 Jena, Germany; Institute of Neuropathology, University Medical Center Hamburg-Eppendorf, 20246 Hamburg, Germany; Institute of Anatomy, Anatomy and Cell Biology, University of Bonn, 53115 Bonn, Germany; Department of Neurology, University Medical Center Hamburg-Eppendorf, 20246 Hamburg, Germany; Department of Oral and Maxillofacial Surgery, University Medical Center Hamburg-Eppendorf, 20246 Hamburg, Germany; Department of Diagnostic and Interventional Radiology, University Medical Center Hamburg-Eppendorf, 20246 Hamburg, Germany; Plymouth University Peninsula Schools of Medicine and Dentistry, Plymouth, Devon PL6 8BU UK; Department of Pediatrics, Herman B Wells Center for Pediatric Research Department of Biochemistry, Indiana University School of Medicine, Indianapolis, IN 46202 USA

**Keywords:** Schwannoma, Neurofibromatosis type 2, NF2, Tumor induction, Microenvironment, Sciatic nerve, Crush injury, tissue inflammation

## Abstract

Schwannomas are predominantly benign nerve sheath neoplasms caused by *Nf2* gene inactivation. Presently, treatment options are mainly limited to surgical tumor resection due to the lack of effective pharmacological drugs. Although the mechanistic understanding of *Nf2* gene function has advanced, it has so far been primarily restricted to Schwann cell-intrinsic events. Extracellular cues determining Schwann cell behavior with regard to schwannoma development remain unknown. Here we show pro-tumourigenic microenvironmental effects on Schwann cells where an altered axonal microenvironment in cooperation with injury signals contribute to a persistent regenerative Schwann cell response promoting schwannoma development. Specifically in genetically engineered mice following crush injuries on sciatic nerves, we found macroscopic nerve swellings in mice with homozygous *nf2* gene deletion in Schwann cells and in animals with heterozygous *nf2* knockout in both Schwann cells and axons. However, patient-mimicking schwannomas could only be provoked in animals with combined heterozygous *nf2* knockout in Schwann cells and axons. We identified a severe re-myelination defect and sustained macrophage presence in the tumor tissue as major abnormalities. Strikingly, treatment of tumor-developing mice after nerve crush injury with medium-dose aspirin significantly decreased schwannoma progression in this disease model. Our results suggest a multifactorial concept for schwannoma formation—emphasizing axonal factors and mechanical nerve irritation as predilection site for schwannoma development. Furthermore, we provide evidence supporting the potential efficacy of anti-inflammatory drugs in the treatment of schwannomas.

**Electronic supplementary material:**

The online version of this article (doi:10.1007/s00401-016-1583-8) contains supplementary material, which is available to authorized users.

## Introduction

Schwannomas are benign Schwann cell-derived nerve sheath tumors that can occur either sporadically—in association with genetic syndromes such as schwannomatosis or neurofibromatosis type 2 (NF2)—or as a result of therapeutic irradiation. The annual incidence of all schwannomas has been estimated at 2.1 per 100,000 people [2]. Furthermore, autopsy studies suggest a prevalence of 4.5 % for sporadic schwannomas in older individuals [44].

In virtually all sporadic schwannomas, as well as in NF2 disease, schwannoma development is genetically caused by mutations in the *Nf2* gene [55], which encodes for the merlin tumor suppressor protein. Although schwannomas are predominantly benign tumors, they can cause a significant decline in life quality of afflicted individuals. Patients may present with pain, loss of sensation, paraesthesia and/or weakness of extremities, depending on the position of the schwannoma and the nerve involved. NF2 disease can often mean lifelong deafness, as schwannomas predominantly appear at the vestibulocochlear nerves (vestibular schwannoma).

Current pharmacological treatment options for schwannomas, e.g., bevacizumab treatment applying anti-VEGF monoclonal antibodies [40], are limited to disease stabilization and can present severe side effects with long-term use [29]. Thus, therapeutic possibilities are often limited to surgical resection or radiosurgery of tumor tissue, with the additional risk of iatrogenic nerve damage. The multifocality of tumors in patients with tumor predisposition syndromes such as NF2, further challenges the practicability of surgical intervention. Hence, an urgent need exists for novel therapeutic approaches and a better understanding of the molecular and cellular mechanisms leading to schwannoma development.

The first animal model in the field bearing a Schwann cell-specific ablation of the tumor suppressor produced NF2-related tumor formation in mice [14]. However, only 24 % of these genetically engineered animals (P0-Cre;Nf2^fl/fl^) spontaneously developed schwannomas after 24 months. In another disease model, conditional *nf2* gene deletion using the Periostin-Cre driver line (Postn-Cre;Nf2^fl/fl^) leads to the appearance of spinal, peripheral and cranial nerve schwannomas in all animals by the age of 10 months [13].

Loss of the merlin tumor suppressor protein, responsible for NF2 disease, has been intensively studied with respect to its ability to act on proliferation, migration, differentiation and tumorigenesis. As a result, merlin has been demonstrated to interact with a variety of different signaling pathways [8]. However, ongoing research mainly focuses on Schwann cell-intrinsic events. Recently, a neuronal function of merlin has been explored in more detail in our laboratory, emphasizing an implication in NF2-related neuropathy [45] as well as in the bi-directional communication of axons and Schwann cells [47]. Specifically, we could show that merlin in neurons has a relevant impact on receptor expression and intracellular signaling events of adjacent Schwann cells, by involving the Nrg1-ErbB pathway [36].

Tumor formation from Schwann cells into schwannomas is mainly thought to be dependent on bi-allelic inactivation of the *Nf2* tumor suppressor gene, in line with Knudson’s two-hit hypothesis [22]. However, loss of heterozygosity (LOH) has reportedly been detected in only 67 % of NF2-related and 56/57 % of sporadic schwannomas [15, 24]. Consistently, 40 % of all schwannomas were found to have at least one intact wild-type copy of the *Nf2* gene remaining [51]. Intronic or promoter-region mutations within the *Nf2* gene, or mutations in genes other than *Nf2,* might lead to an overestimation of these numbers. Nonetheless, it appears unresolved whether inactivation of both the *Nf2* alleles in Schwann cells is a prerequisite for schwannoma development. Mechanistic and experimental studies resolving this issue are not currently available. In addition, it is not understood whether LOH occurs as early as in microscopic schwannoma precursors, referred to as tumorlets [49].

Where and why schwannomas ultimately appear also remains to be fully understood. Throughout life, most peripheral nerves are likely to be injured repetitively, due to compressive events and mechanical irritations. During the course of physiological nerve regeneration in the peripheral nervous system, Schwann cells lose contact with injured axons—thereby allowing them to de-differentiate and proliferate again. However, in cases of pathological cell-to-cell communication, Schwann cells cannot properly align to axons and remain de-differentiated with continuous proliferation [35, 39]. Axons are therefore thought to be essential for maintaining Schwann cells in a differentiated state during adulthood.

Based on our previous findings indicating the importance of merlin in axon–Schwann cell signaling events [47], we hypothesized that genetically defined alterations of the nerve microenvironment could contribute to the initial events of schwannoma development [48]. Specifically, we aimed to investigate in mice whether the loss of just one merlin allele in both the axonal and Schwann cell compartment of peripheral nerves, is sufficient to provoke long-lasting regenerative abnormalities when challenged by an injury of the sciatic nerve. Another aim of this study was to improve the understanding of schwannoma biology with respect to minimal nerve trauma, which might predispose the lesion sites to enhanced Schwann cell proliferation.

## Materials and methods

### Experimental animals

All mice used in this study were handled in strict adherence to local governmental and institutional animal care regulations. Animals had free access to food and water and were housed under constant temperature and humidity conditions on a 12/12-h light/dark cycle. The following transgenic mouse strains were used for the study: Nf2flox animals (RIKEN BioResource Centre) were used to obtain conditional Schwann cell-specific merlin knockout by crossing with the P0-Cre line (The Jackson Laboratory, USA, stock 017928). In order to achieve neuron-specific loss of merlin, we mated Nf2flox animals with a mouse strain that expresses Cre recombinase under the neurofilament heavy class promoter (Nefh-Cre) (The Jackson Laboratory, stock 009102). Furthermore, we generated animals showing double Cre expression by breeding Nf2flox;P0-Cre mice with Nf2flox; Nefh-Cre animals (Nf2flox;P0-Cre;Nefh-Cre). Cre recombinase-specific genotyping was performed using the following primers: 5′-CCA CCA CCT CTC CAT TGC AC-3′ (forward) and 5′-ATG TTT AGC TGG CCC AAA TG-3′ (reverse) for P0-Cre [12], as well as 5′-GGG CCA CCG CGG ATA TAA AA-3′ (forward) and 5′-TGC GAA CCT CAT CAC TCG TT-3′ (reverse) for Nefh-Cre recombinase [17]. Mice bearing a complete heterozygous merlin knockout were generated by crossing Nf2flox animals with Amh-Cre mouse line (The Jackson Laboratory, USA, stock 007915). This line expresses Cre recombinase driven by the Sertoli cell-specific promoter of the anti-Mullerian hormone (Amh) gene. Offsprings carry *nf2* gene knockout in the germline (Nf2^Δ/+^) and spontaneously develop hepatocellular carcinoma, amongst other previously described tumor entities [30]. All animals and corresponding wild-type littermates were on a mixed C57BL/6-FVB/N background. Both genders were used in equal numbers in this study.

### Sciatic nerve crush injury

Unilateral injuries of sciatic nerves were accomplished according to a previously described method [4]. Briefly, 8- to 10-week-old mice were anesthetized using 2 % isoflurane in 100 % oxygen. Fur was then removed from one hind limb. After an appropriate incision of the skin, the gluteal musculature was separated in order to reveal the right sciatic nerve. Using hemostatic forceps (Ultra Fine Haemostat, #13021-12, tip width 0.6 mm, Fine Science Tools, Germany), the nerve was crushed once by the application of a defined pressure for 20 s. The locking mechanism of the hemostatic forceps with a series of interlocking teeth ensured reproducibility and standardization of crush injury. Finally, both the gluteal musculature and skin incision were sutured using non-absorbable surgical suture material (Polyethylene terephthalate; USP 4/0, #17218113, Catgut GmbH, Germany).

### In vivo magnetic resonance imaging

Magnetic resonance imaging was performed with a dedicated small animal MR scanner at 7.0 T (ClinScan 70/30, Bruker, Germany). The scanner is equipped with a high-performance gradient system allowing maximal gradient amplitude of 630 mT m^−1^ and a slew rate of up to 6300 T m^−1^ s^−1^. The data were acquired using a birdcage polarized transmit/receive coil with an inner diameter of 40 mm.

The MR sequence protocol consisted of a T1-weighted localizer sequence in three orthogonal planes, followed by a high-resolution fat-saturated T2-weighted 2D turbo spin-echo (TSE) sequence in coronal, sagittal and axial orientation. Image parameters of the coronal and sagittal T2 TSE sequence were as follows: time of repetition (TR)/time of echo (TE)/flip angle (FA) = 4000 ms/47 ms/90°; field-of-view (FOV) = 90 × 36 mm^2^; matrix = 512 × 208; slice thickness (ST) = 0.8 mm, number of slices (NS) = 16; number of acquisitions (NA) = 4; spatial resolution = 0.17 × 0.17 × 0.8 mm; acquisition time = 3 min 57 s. Image parameters of the fat-saturated axial T2 TSE sequence were as follows: TR/TE/FA = 2362 ms/90 ms/90°; FOV = 32 × 32 mm^2^; matrix = 256 × 256; ST = 0.6 mm, NS = 19; NA = 3; spatial resolution = 0.12 × 0.12 × 0.6 mm; acquisition time = 9 min 26 s.

### Neuropathological assessment and evaluation

For the histological workup, paraformaldehyde-fixed nerve samples were embedded in paraffin, cut at the site of the largest diameter and mounted as a tissue microarray. Four-micrometer thick cross sections were used for H&E staining and immunohistochemical labeling of S100 (1:8000, Dako, Germany), myelin protein zero (P0) (1:300, Bioss Antibodies, USA), phospho-c-Jun (Ser73, 1:500, Cell Signaling, USA, 8752), ErbB2 (1:500, Cell Signaling), p75 (1:200, Millipore, USA, AB1554), Sox10 (1:200, Abcam, UK, ab155279**),** MMR/CD206 (1:200; R&D Systems; AF2535) and Arginase-1 (1:200, Santa Cruz, USA, H-52) in an automated Ventana stainer (Ventana Medical Systems, USA) using standard antigen retrieval protocols (CC1st, no pre-treatment for S100 protein). Onion bulbs and schwannoma-like structures were assessed in H&E-stained slides.

### Human schwannoma samples

The investigation of human schwannomas was performed using a tissue microarray comprising 30 sporadic schwannomas, 10 NF2-associated schwannomas and four schwannomatosis-associated schwannomas. The tissue microarray was built from larger paraffin-embedded tumor specimens that were first reviewed by a neuropathologist. Appropriate tumor areas were chosen and two tissue cylinders 1 mm in diameter were punched out and arranged in a new paraffin block. Labeling of CD68 (1:100, Dako, #M0876, no pre-treatment), MMR/CD206 (1:200; R&D Systems; AF2535) and Iba-1 (1:200, Wako, Japan) was carried out in an automated Ventana stainer. Bound antibodies were detected by the peroxidase method, using diaminobenzidine as chromogen (Ventana Medical Systems, #760-500). Labeling intensities were evaluated semi-quantitatively.

### Immunohistochemistry and cell quantification

Immunohistochemistry was performed as described in [46]. Briefly, paraffin-embedded longitudinal sections of sciatic nerves were rehydrated, boiled in 10 mM sodium citrate buffer (pH 9) for 30 min in a microwave and subsequently treated with 0.5 % Triton X-100 for 10 min. Sections were then incubated in 0.2 % gelatin and 2 % goat serum diluted in PBS for at least 2 h, followed by submersion in the primary antibody solution overnight at 4 °C. The following primary antibodies were used: P0 (1:200, Abcam, UK, ab39375), neurofilament (1:200, BioLegend, USA, SMI312), myelin basic protein (MBP) (1:500, Millipore, USA, MAB384), Ki-67 (1:200, eBioscience, USA, clone SolA15), Iba-1 (1:200, Wako, Japan) and p75 (1:200, Millipore, USA, AB1554). After vigorous washings, sections were incubated with the secondary antibody solution (Alexa488-, Alexa546- or Alexa647-conjugated anti-mouse, -rat, -chicken and -rabbit antibodies (1:500 in PBS, Invitrogen, USA) at room temperature for 2 h. Finally, specimens were washed in PBS, counterstained using DAPI (1 µg/ml PBS, 10 min), dehydrated and embedded. DAPI-stained cell nuclei or Iba-1-positive cells were counted using ImageJ plug-in ‘Particle Analysis’, from images acquired with 10× magnification after standardized background reduction and threshold setting.

### Immunoblotting

Immunoblotting was performed as described in [32]. Separate pooled protein lysates were prepared from both intact and crushed sciatic nerves. The following primary antibodies were used: phospho-c-Jun (Ser73, 1:1000, Cell Signaling, USA, 8752), Neuregulin 1 (1:250, Santa Cruz, USA, clone C-20), Erk (1:500, Cell Signaling, USA), phospho-Erk (T202/Y204, 1:500, Cell Signaling, USA), ErbB2 (1:500, Cell Signaling, USA), GAPDH (1:1000, Santa Cruz, USA, 6C5) and merlin (1:500, Santa Cruz, USA A19). Results were quantified using gel analysis software by ImageJ. Density values were normalized to GAPDH and appropriate controls of transfection or wild-type tissue, respectively. In the case of phospho-specific detection of proteins, their acquired densities were referred to signals derived from related pan-antibodies that served as loading control (e.g., phospho-Erk to Erk signals).

### Morphometric analysis of nerve sections

Analysis of axon caliber, myelination thickness and solidity factor was conducted on semi-thin sections of sciatic nerves removed from transcardially perfused mice. Mice were perfused with a solution containing 3 % paraformaldehyde and 3 % glutaraldehyde in 0.1 M phosphate buffer (pH 7.4). Sections obtained from the distal part of the sciatic nerve were post-fixed for 1 h and kept in fixative including 3 % sucrose. Images of toluidine blue-stained semi-thin cross sections were taken using an Axioskop 2 MOT (Carl Zeiss, Germany) equipped with a 100× immersion oil objective and an Olympus XC50 digital camera (Olympus, Germany). Standardized settings for camera sensitivity, resolution (2576 × 1932 pixels) and brightness of illumination were used for all micrographs. Image analysis was conducted with ImageJ version 1.48u. RGB color images obtained from semi-thin sections were split into single channels and the green channel was chosen for measurements. The image was contrasted using the auto-function. Axon and myelin were circumscribed manually by the freehand selection tool. Based on the measured areas, the thicknesses of the axons and myelin sheaths were calculated. The solidity factor is a parameter for quantifying the roundness of axons [45] and describes the area covered by a given structure in relation to the smallest convex area, which covers this structure; that is, the solidity factor of a round circle or ellipse would be 1, whereas a circle with an invagination would give a factor smaller than 1. This solidity factor is superior to the circularity factor often used for measuring changes in surface structures, as it provides a factor to measure changes in surface curvatures independent of the plane of section. All calculations and statistics were done in R (http://www.r-project.org/).

### Electron microscopic evaluation of nerves

For the preparation of ultra-thin sections of sciatic nerves, mice were perfused with a solution containing 2 % paraformaldehyde and 2.5 % glutaraldehyde (vol/vol) in 0.1 M cacodylate buffer (pH 7.2). Tissue was post-fixed for 6 h and then kept in fixative that included 3 % sucrose (vol/vol). Sections were cut from the mid part of the sciatic nerve close to the crushed area and stained with gadolinium acetate and lead citrate. Images were taken with a Zeiss Libra 120.

### LOH analysis of nerve specimens

Simple repeats were identified by screening the sequence of the genomic *nf2* region on mouse chromosome 11 (4765845 to 4849536 in GRCM38) using a web tool [28]. Flanking primers were designed in intron 5 of the *nf2* gene. One primer was labeled with FAM at the 5′-end. The polymorphic marker was amplified from three pooled nerves of wild type and P0-Cre;Nefh-Cre;Nf2^fl/+^ mice, respectively, by means of polymerase chain reaction (PCR), for a total of 30 cycles at an annealing temperature of 60 °C. The amplified marker was analyzed on a sequencer ABI310. As the amplification of polymorphic markers is PCR-based, the resolution of this standard method is naturally limited. Our unpublished data demonstrate that if an LOH is present in less than 25 % of cells in a sample, it will not be reliably detected (Kluwe 2016, manuscript submitted).

### ASS treatment

Acetylsalicylic acid (ASS; aspirin) was purchased from Sigma-Aldrich (A5376; USA), firstly resolved in DMSO and further diluted in phosphate-buffered saline (PBS) to reach target concentration. For systemic administration, 5 mg aspirin per kg body weight was intraperitoneally injected into mice every other day (5 mg aspirin per kg body weight in mice equates to approximately 375 mg dosage for humans). PBS solution without ASS served as vehicle control.

### In situ tumor size quantification

The right sciatic nerve of anesthetized animals was exposed surgically in order to assess sciatic nerve diameter as indicator for tumor size. Documentation was performed by video-assisted microscopy for each animal. Selected frames from the video files (using VirtualDub 1.6.19 software) were used to determine maximal sciatic nerve diameter using ImageJ.

### Mouse cytokine detection in nerve lysates

For the unbiased detection of 40 different cytokines from nerve tissue, a commercially available kit was used according to the manufacturer’s instructions (Mouse Cytokine Array Panel A, R&D Systems, USA). Pooled total nerve lysates (150 μg each), taken 8 months after crush injury from four animals per genotype, were used for this ELISA-based method. The chemiluminescence of the membrane was analyzed by pixel density quantification using ImageJ. The relative changes of cytokine levels between samples were subsequently calculated.

### Statistical analysis

Comparisons between groups were done using unpaired* t* test or one-way analysis of variance (one-way ANOVA) with subsequent Tukey’s multiple comparisons test (TMCT) for the comparison of multiple groups (SPSS software, Statistical Package for the Social Sciences, USA). For each experiment, we calculated the *p* value (*P*). Differences were considered significant when *P* < 0.05. All values are presented as means and the corresponding standard errors.

## Results

### Nerve crush injury induces macroscopic nerve swelling

Based on our previous findings on the importance of merlin for axon-to-Schwann cell signaling events [47], we hypothesized that genetically defined alterations of the nerve microenvironment would also contribute to the initial events of schwannoma development. To that end, we generated conditional knockout mouse lines bearing cell type-specific deletion of merlin in neurons and/or Schwann cells (Supplementary Fig. 1). However, neither spontaneous schwannoma development nor obvious nerve swellings were observed in any of the mouse lines within the first 10 months of lifespan (data not shown). Nevertheless, examination of semi-thin cross sections of sciatic nerves from 10-month-old mice (Supplementary Fig. 2) and subsequent morphometric analysis of nerve parameters, revealed a significant axon diameter decrease in nerves taken from P0-Cre;Nf2^fl/fl^ and P0-Cre;Nefh-Cre;Nf2^fl/+^ mice (Supplementary Fig. 3); as well as reduced roundness of axons (solidity factor) in animals bearing a neuron- and/or Schwann cell-specific loss of the *nf2* gene (Supplementary Fig. 4). Furthermore, we could detect differences in myelin thickness and the g-ratio (Supplementary Fig. 5 and 6). In sum—despite remarkable changes in nerve fine structure—spontaneous schwannoma growth could not be observed in the investigated mouse lines. In particular, the nerve structure of P0-Cre;Nefh-Cre;Nf2^fl/+^ mice was found to be significantly disrupted (Supplementary Fig. 2).

**Fig. 1:**
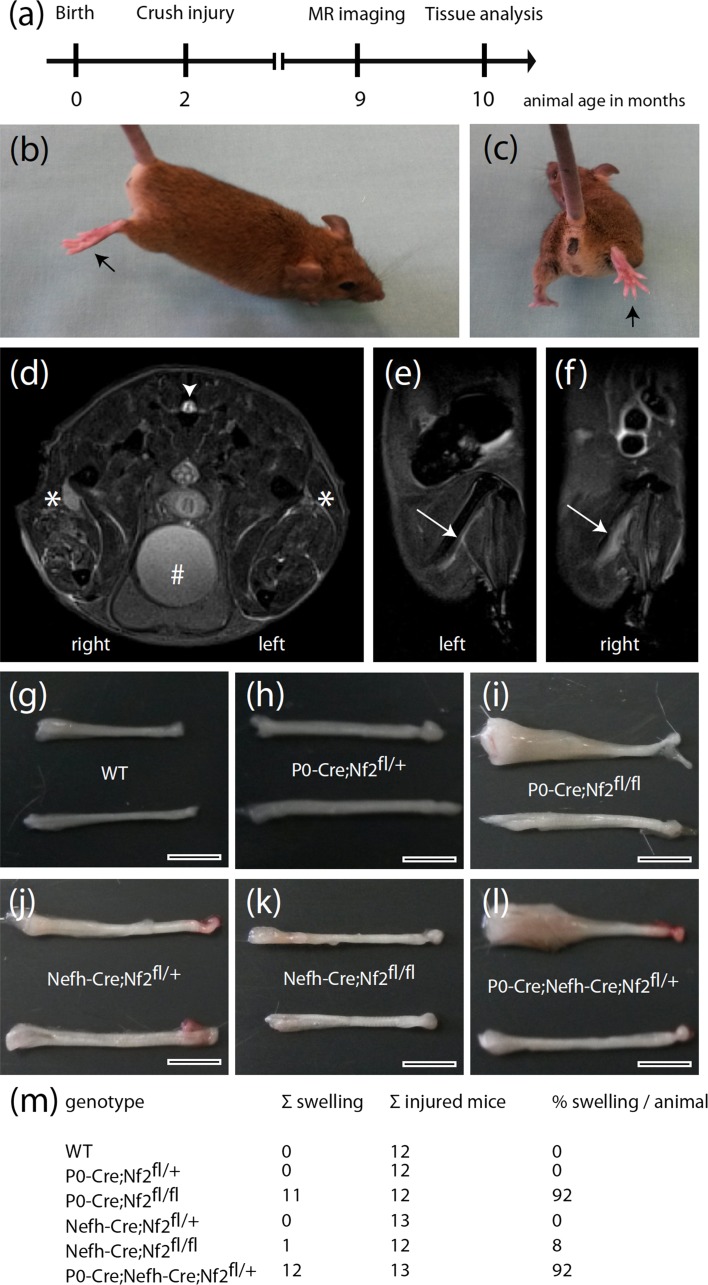
Macroscopic analysis of nerve morphology 8 months after crush injury. **a** Schematic representation of the study protocol. **b**, **c** Representative images of one mouse 8 months after crush injury, indicating a relieving posture of the right hind limb (*arrow*) bearing the injured sciatic nerve when gently lifted at its tail. **d**–**f** T2-weighted coronal (**d**) and sagittal (**e**, **f**) MR images from a P0-Cre;Nefh-Cre;Nf2^fl/+^ mouse 7 months after sciatic nerve crush. **d** *Asterisks* show the position of the *left* (uninjured) and *right* (crushed) sciatic nerve. *Arrowhead* indicates the spinal cord and hash marks the urine-filled bladder. *Arrow* in **e**, **f** depicts the anatomical course of the sciatic nerve close to the femur. The injured *right* sciatic nerve shows enlargement compared to the non-injured sciatic nerve on the *left side*. **g**–**l** Dissected sciatic nerves from 10-month-old mice of indicated genotypes are shown. Crushed nerves (8 months post-injury) are shown at the *top*; intact nerves are depicted at the *bottom* of each representative image. Proximal nerve parts are on the *right side* of the images; distal parts on the *left side*. *Scale bars* represent 3 mm. **m** Quantification of macroscopic nerve swellings following nerve crush injury. A nerve swelling was counted when the nerve radius was at least doubled

We therefore decided to challenge the pre-existing nerve alterations due to conditional merlin knockout (Supplementary Fig. 1), by performing a unilateral crush injury to the right sciatic nerve of 2-month-old mice (Fig. 1a). We hypothesized that a combination of genetically defined conditions of the nerve microenvironment and regenerative processes could provoke schwannoma formation in vivo. In fact, cell proliferation following tissue injury normally subsides after the assaulting factor is removed, or the repair completed. But proliferating cells that have pre-existing mutations [43], or acquire DNA damage, continue to proliferate in microenvironments rich in inflammatory cells [10].

Crush injuries were chosen in favor of cut injuries, because crushes better mimic a condition of mild nerve traumas that can occur during the lifetime of human individuals. All mice were examined regularly for behavioral abnormalities during the months that followed the sciatic nerve injury. In this respect, none of the animals displayed obvious walking difficulties or a compromised general state of health. However, upon closer inspection—when gently lifted at their tail—animals with the homozygous merlin deletion in Schwann cells (P0-Cre; Nf2^fl/fl^) and the combined heterozygous knockout in Schwann cells and neurons (P0-Cre;Nefh-Cre;Nf2^fl/+^), presented with a relieving posture of the right hind limb bearing the injured sciatic nerve (Fig. 1b, c). Seven months after crush injury, a subset of mice was analyzed for nerve swellings using in vivo magnetic resonance imaging (MRI). In axial T2-weighted images, both P0-Cre;Nf2^fl/fl^ and P0-Cre;Nefh-Cre;Nf2^fl/+^ animals showed remarkable volume gain of the injured right sciatic nerve when compared to the unaffected left sciatic nerve (Fig. 1d). Sagittal T2-weighted images depicting the nerve anatomy in more detail, further revealed long-segment unilateral swelling of the right sciatic nerve (Fig. 1e, f). Eight months after the crush injury, sciatic nerve tissue from all animals were dissected and collected. In order to assess macroscopic swelling, previously injured sciatic nerves were compared to unaffected nerves of the contralateral side (Fig. 1g–l). In summary, over 90 % of animals with the homozygous merlin deletion in Schwann cells (P0-Cre;Nf2^fl/fl^) and the combined heterozygous knockout in Schwann cells and neurons (P0-Cre;Nefh-Cre;Nf2^fl/+^) presented with marked nerve swelling 8 months after nerve crush injury (Fig. 1m). In contrast, the other genotypes showed no comparable gain in nerve thickness.

### Neuropathological assessment of crush-induced sciatic nerve tumors

**Fig. 2:**
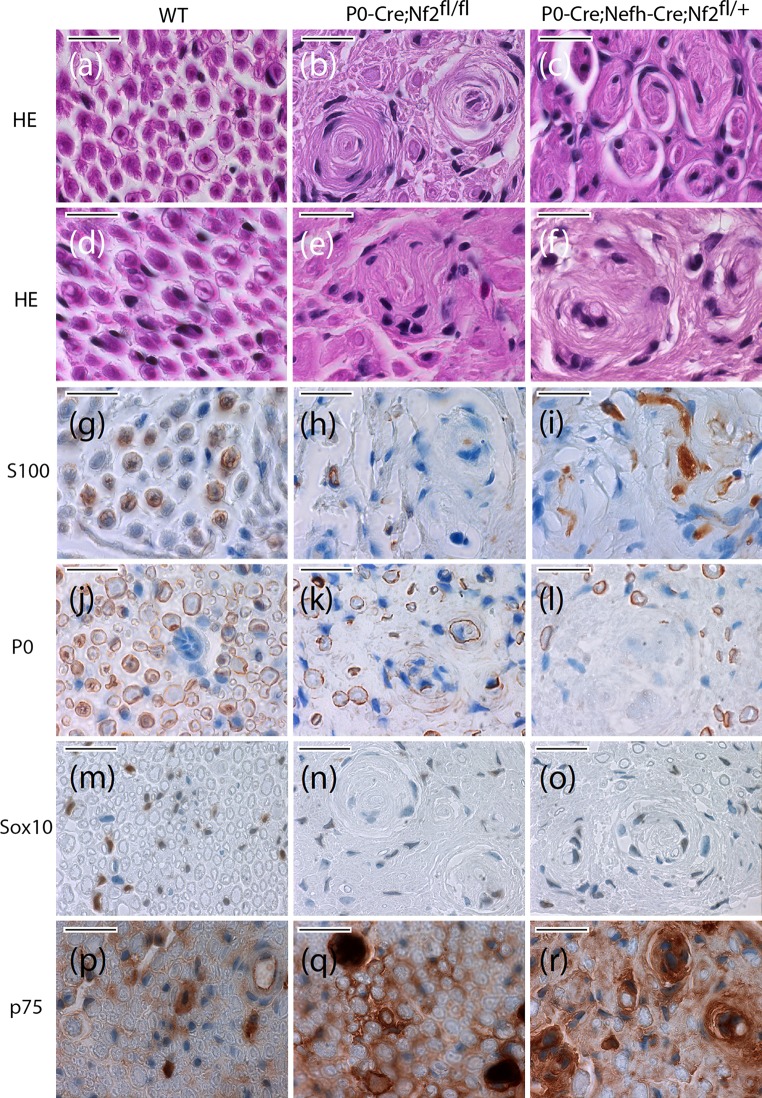
Appearance of schwannoma-like structures and tumorlets in nerves from P0-Cre;Nefh-Cre;Nf2^fl/+^ mice. **a**–**l** Sciatic nerve cross sections of indicated genotypes 8 months after crush injury were either HE stained (**a**–**f**), or immunolabeled (*brown color*), for Schwann cell markers S100 (**g**–**i**) and P0 (**j**–**l**), as well as neural-crest marker Sox10 (**m**–**o**) and p75 as indicator for immature non-myelinating Schwann cells (**p**–**r**). Cell nuclei are visualized in *blue*. *Scale bar* represents 20 μm

Focusing on the two genotypes that did develop macroscopic nerve swelling (P0-Cre;Nf2^fl/fl^ and P0-Cre;Nefh-Cre;Nf2^fl/+^), nerve cross sections were analyzed neuropathologically 8 months after crush injury. Wild-type animals showed normal nerve composition after regeneration (Fig. 2a, d), as well as regular expression of Schwann cell marker S100 (Fig. 2g) and P0 as indicator for Schwann cell differentiation in the myelin sheath (Fig. 2j). Both the neural crest lineage marker Sox10 (Fig. 2m) and the p75 neurotrophin receptor (marker for immature non-myelinating Schwann cells) showed an expected staining pattern (Fig. 2m) [19, 21].

In contrast, injured nerves of P0-Cre;Nf2^fl/fl^ animals bearing the homozygous merlin deletion in Schwann cells demonstrated imperfect regeneration, with large concentric multilayered onion bulbs indicative of for repetitive de- and re-myelination processes (Fig. 2b), as well as sporadic small disordered Schwann cell clusters (Fig. 2e). S100 protein expression (Fig. 2h) and P0 expression (Fig. 2k) appeared to be decreased, in comparison to wild-type mice. While Sox10 immunoreactivity was normal (Fig. 2n), p75 stainings suggest the presence of more immature non-myelinating Schwann cells (Fig. 2q).

In P0-Cre;Nefh-Cre;Nf2^fl/+^ animals, regeneration of sciatic nerves 8 months after crush injury was found to be even more disturbed, but onion bulbs appeared smaller and less structured (Fig. 2c) than in P0-Cre;Nf2^fl/fl^ mice. Instead, larger clusters of unordered Schwann cells were detected, resembling tumorlets (microscopic neoplastic Schwann cell proliferations) observed in nerves of NF2 patients (Fig. 2f and Supplementary Fig. 7). Like tumorlets in sural nerve biopsies of NF2 patients [16], the disordered cell clusters presented an Antoni A growth pattern, the primary growth pattern of schwannoma in mice [50]. Focally increased S100 protein expression was observed within Schwann cell clusters (Fig. 2i), whereas P0 as a marker of differentiation was not detectable in several regions of the tissue (Fig. 2l). Sox10 expression, however, indicates the presence of neural crest-derived cells (Fig. 2o), in combination with increased cell immaturity, as suggested by increased p75 immunoreactivity (Fig. 2r).

In summary, both genotypes that developed sciatic nerve swelling 8 months after crush injury showed neuropathological abnormalities, with the pattern more disordered in animals with the combined heterozygous *nf2* gene deletion in Schwann cells and the neuronal compartment of peripheral nerves (P0-Cre;Nefh-Cre;Nf2^fl/+^). It was only in these haploinsufficient animals with reduced *nf2* gene dosage, that Schwann cell clusters resembling NF2 patient tumorlets were observed. Furthermore, as in human schwannomas, S100 protein expression was up-regulated while P0 expression was lost [18].

**Fig. 3:**
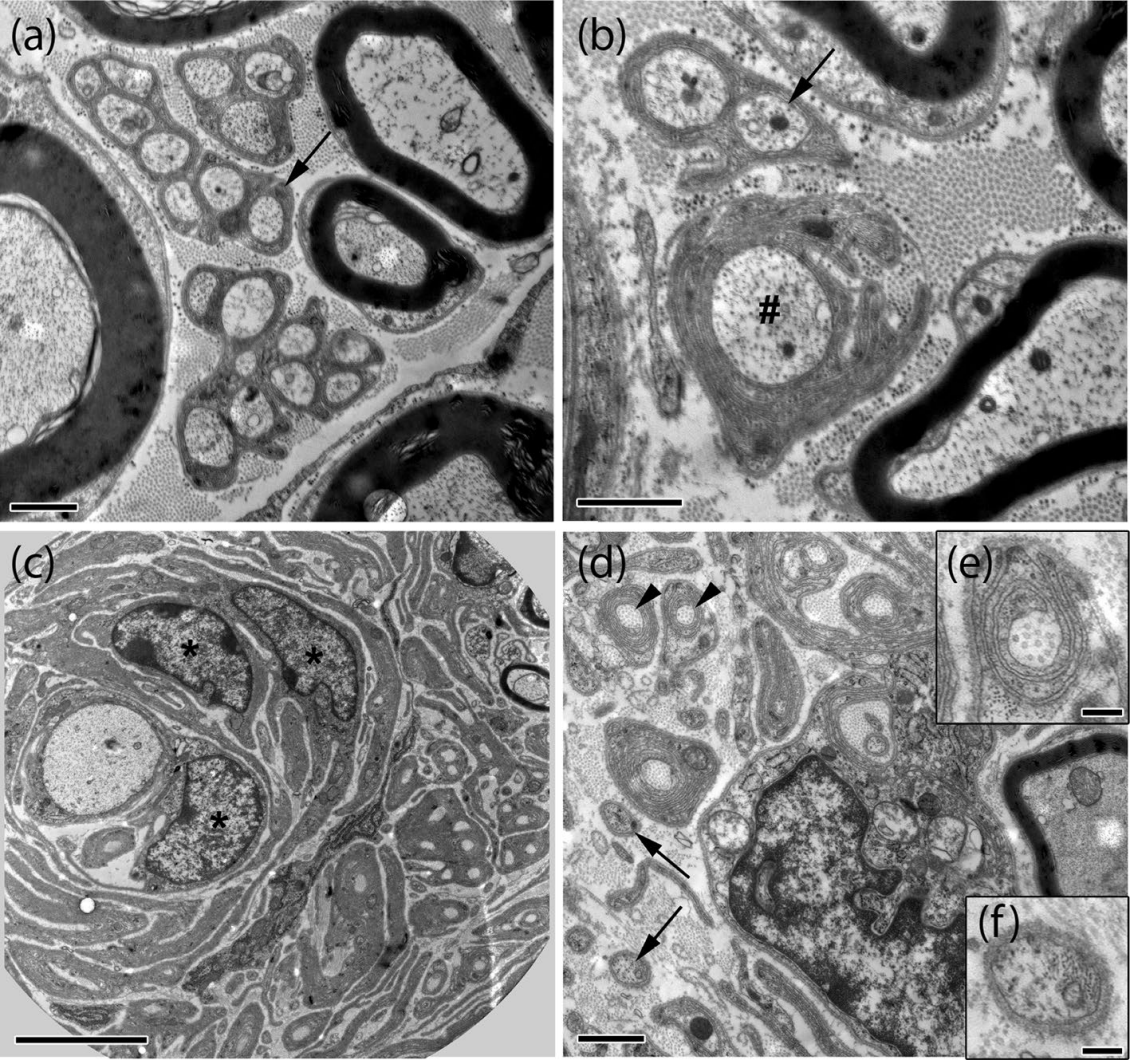
Electron microscopy reveals multiple signs of degeneration in crushed and uncrushed nerves of P0-Cre;Nefh-Cre;Nf2^fl/+^ mice. Representative electron microscopic images of intact (**a**, **b**) and crushed (**c**–**f**) sciatic nerves (8 months post-injury) taken from P0-Cre;Nefh-Cre;Nf2^fl/+^ mice. While myelinated axons and Remak bundles of various size (*arrows* in **a**, **b**) are frequent in intact nerves, myelinated axons and Remak bundles appear rarely in crushed nerves (**c**, **d**). Non-myelinated axons were mostly seen as single axons (*arrows* in **d**, **f**). Signs of degeneration such as collagen pockets (*arrowhead* in **d**, **e**), abnormally thin myelin sheath and layers of Schwann cell processes enwrapping axons (**c**), bands of Bungner and axons with irregular myelin sheaths (*hash* in **b**) were mostly present in crushed, but, to a minor extent, also in intact nerves. In addition, accumulations of Schwann cells could be observed (*asterisks* in **c**). *Scale bars* in **a**, **b**, **d** represent 1 μm. *Scale bar* in **c** 5 μm. *Scale bars* in **e**, **f** represent 0.2 μm

Further ultrastructural analysis of sciatic nerve tissue from 10-month-old P0-Cre;Nefh-Cre;Nf2^fl/+^ mice confirmed multiple signs of degeneration in intact (non-crushed) nerves (Fig. 3a, b; Supplementary Fig. 2). But even more prominently, tumorlet-containing nerves appear structurally disordered exhibit concentric layers of Schwann cell processes and collagen around axons (onion bulbs) 8 months after crush injury (Fig. 3c–f).

### Nerve regeneration defect in mice with nerve swellings after crush injury

**Fig. 4:**
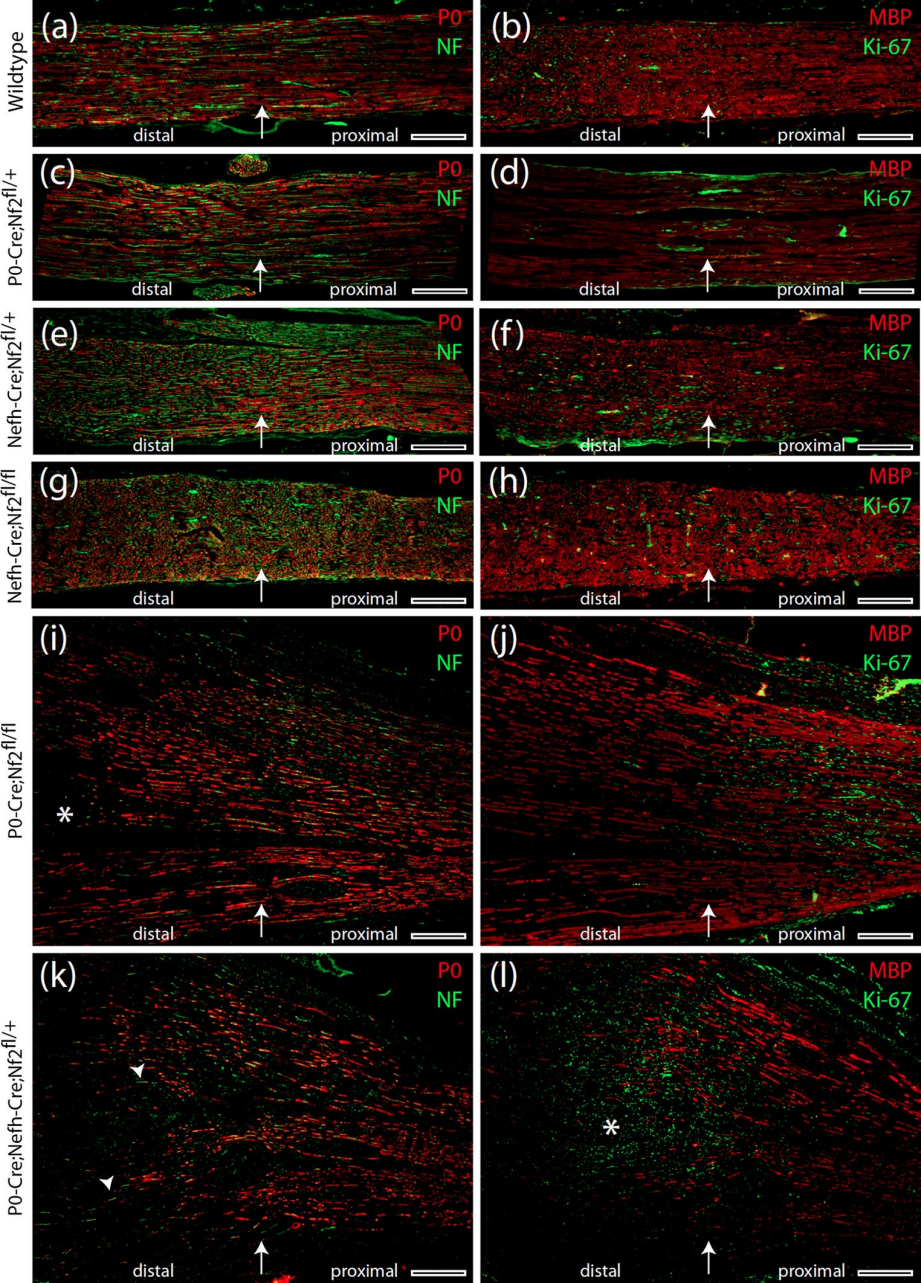
Severe re-myelination defect in P0-Cre;Nefh-Cre;Nf2^fl/+^ mice following nerve crush. **a**–**l** Immunohistochemical stainings of longitudinal sciatic nerve sections prepared from indicated genotypes 8 months after crush injury. Immunolabeling of P0, neurofilaments, MBP and Ki-67 indicates Schwann cell differentiation, axonal fibres, myelination and cell proliferation, respectively. *Arrow* in each image shows the position of the nerve crush. Orientation of nerves is stated as ‘distal’ and ‘proximal’. *Asterisk* in **i** emphasizes an area of defective re-myelination. *Arrowheads* in **k** indicate axons devoid of any myelin sheath. *Asterisk* in **l** marks concentration of Ki-67-positive cells at the edge of intact myelination. *Scale bars* represent 200 μm

In order to further explore detailed nerve anatomy 8 months after crush injury, we performed immunohistochemistry on longitudinal nerve sections covering the lesion site as well as the adjacent proximal and distal parts. We used fluorescent labeling of P0 as a marker for Schwann cell differentiation and myelin basic protein (MBP) as indicator for myelination as well as of neurofilaments for axonal tracing. By this means we found that both axon integrity and myelination continuity were preserved in nerves from P0-Cre;Nf2^fl/+^ (Fig. 4c, d), Nefh-Cre;Nf2^fl/+^ (Fig. 4e, f) and Nefh-Cre;Nf2^fl/fl^ (Fig. 4g, h) animals, as well as in their wild-type littermates (Fig. 4a, b). Immunostaining for Ki-67—a well-established indicator of cell proliferation—shows a patchy distribution in these nerves (Fig. 4b, d, f, g) without an obvious focus with regard to the crush site.

In P0-Cre;Nf2^fl/fl^ animals, the macroscopically swollen nerves showed distinct areas of pathological regeneration, as indicated by missing Schwann cell differentiation (Fig. 4i). Overall Schwann cell differentiation and axon continuity, however, were preserved in crushed P0-Cre;Nf2^fl/fl^ nerves (Fig. 4i, j). Ki-67 expression appeared locally restricted to the proximal, but not distal, part of the crushed nerve in relation to the wound site.

Strikingly, crushed nerves taken from P0-Cre;Nefh-Cre;Nf2^fl/+^ animals displayed a severe re-myelination defect, beginning at the position of the experimental crush, with an overall disorganized pattern of remaining MBP-positive, myelinated axons and the appearance of axons devoid of myelination (Fig. 4k). As regular myelination stopped at the crush site, a prominent increase in proliferating cells (Ki-67 staining) was present at the border between preserved myelination and insufficient re-myelination (Fig. 4l). Furthermore, p75-positive cells indicating immature non-myelinating Schwann cells [43] were most abundantly expressed in sciatic nerve sections from P0-Cre;Nefh-Cre;Nf2^fl/+^ mice (Supplementary Fig. 8).

### Signaling and protein expression changes in sciatic nerve lysates

**Fig. 5:**
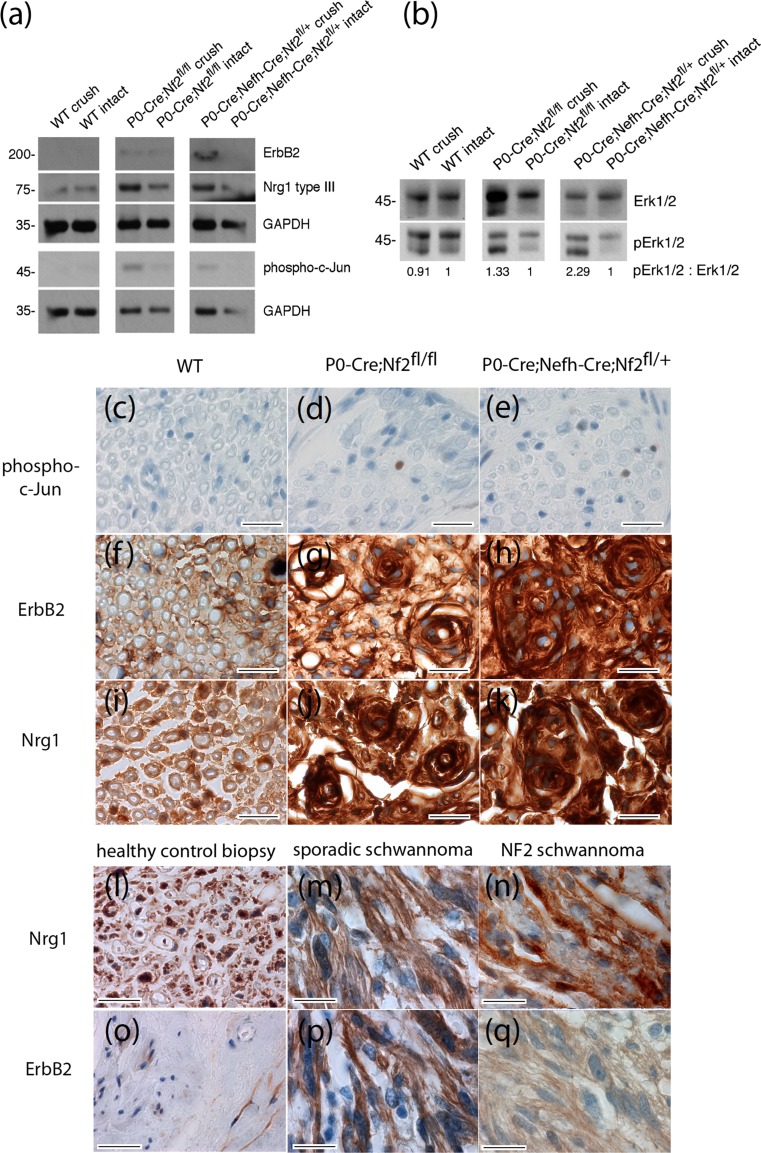
Signaling and protein expression changes in sciatic nerve lysates. **a**, **b** Immunoblot of sciatic nerve lysates (pooled tissue from at least three different animals per indicated genotype was prepared from crushed and intact sciatic nerves 8 months after crush injury). **a** Immunoblot for receptor tyrosine kinase ErbB2, Neuregulin 1 type III (Nrg1 type III), phospho-c-Jun and GAPDH as loading control (*n* = 3). For full-length blot see Supplementary Fig. 9. **b** Immunoblot for phospho-Erk1/2 (pErk1/2). Total protein amount of Erk1/2 served as loading control. Densitometric quantification of pErk1/2: Erk1/2 ratio was normalized to intact nerve tissue for each genotype (*n* = 3). The observed increase in total Erk after crush injury in some genotypes (see also Supplementary Fig. 10) remains unexplained but does not effect the ratio between phospho-Erk and overall Erk. For full-length blot see Supplementary Fig. 10. **c**–**k** Sciatic nerve cross sections of indicated genotypes 8 months after crush injury were immunolabeled (*brown color*) for phospho-c-Jun, as a marker of cellular de-differentiation (**c**–**e**), ErbB2 (**f**–**h**) and Neuregulin 1 (**i**–**k**). Cell nuclei are visualized in blue. *Scale bars* represent 20 μm. **l**–**q** Human tissue sections taken from healthy sural nerve biopsies, as well as sporadic and NF2-associated schwannomas, were immunolabeled (*brown color*) for Neuregulin 1 (**l**–**n**) and ErbB2 (**o**–**q**). Cell nuclei are visualized in *blue*. *Scale bars* represent 20 μm

We next aimed to decipher characteristic signaling pathways relevant to Schwann cell biology in our mouse model for schwannoma development. Using protein lysates from crushed and contralateral intact nerves of conditional knockout animals, we found that the expression of ErbB2, one of the most abundantly expressed receptor tyrosine kinases in human schwannomas [5], was up-regulated in P0-Cre;Nf2^fl/fl^ mice and even more prominently so in P0-Cre;Nefh-Cre;Nf2^fl/+^ animals (Fig. 5a and Supplementary Fig. 9). In line with previous observations from our group on intact mouse nerves [47], the post-cleavage product [56] of the axonal surface molecule Neuregulin1 type III (Nrg1 type III) showed reduced expression in nerves with a reduced amount of merlin protein in the neuronal nerve compartment (Fig. 5a and Supplementary Fig. 9). In essence, we observed a persistent de-regulation of the Neuregulin1-ErbB pathway in both mouse lines that developed macroscopic nerve swellings after nerve injury.

Downstream of receptor tyrosine kinase signaling fine-tuning of the Ras-MAPK pathway is pivotal to resident Schwann cells in preventing tumorigenic transformation [33]. We therefore assessed the phosphorylation-dependent activation of the signaling component Erk1/2, as proxy for the Ras-MAPK pathway in nerve lysates of 10-month-old mice. By relating phospho-specific immunoblot signals to total protein amounts, phosphorylation of Erk1/2 in sciatic nerve lysates was found to be enhanced upon crush in P0-Cre;Nf2^fl/fl^ animals and even more markedly in P0-Cre;Nefh-Cre;Nf2^fl/+^ mice, compared to intact control nerves of each genotype (Fig. 5b and Supplementary Fig. 10).

Phospho-c-Jun expression, indicating Schwann cell immaturity [38], was also slightly increased in crushed nerves from mice with the homozygous *nf2* knockout in Schwann cells (P0-Cre;Nf2^fl/fl^) and in P0-Cre;Nefh-Cre;Nf2 ^fl/+^ mice (Fig. 5a and Supplementary Fig. 9). Consistently, phospho-c-Jun immunolabeling on sciatic nerve cross sections could be detected as a rare event in P0-Cre;Nf2^fl/fl^ (Fig. 5d) and P0-Cre;Nefh-Cre;Nf2^fl/+^ mice (Fig. 4e), when compared to wild-type animals (Fig. 5c). Furthermore, the receptor tyrosine kinase ErbB2 showed highest expression after crush injury in P0-Cre;Nefh-Cre;Nf2^fl/+^ mice (Fig. 5f–h). Nrg1 immunolabeling on sciatic nerve cross sections also appeared similar to the immunoblot results, although the applied Neuregulin 1 antibody cannot distinguish the different types of Nrg1 protein in immunohistochemistry (Fig. 5i–k). In human schwannomas, ErbB2 (Fig. 5l–n) and Nrg1 (Fig. 5o–q) expression was found to be similarly changed compared to sural nerve biopsies from healthy human individuals.

In summary, we could detect clear signaling and protein expression changes as a consequence of both merlin knockout and crush injury. The most dramatic changes in terms of ErbB2 receptor upregulation and Ras-MAPK pathway activation were found in nerve tissue of P0-Cre;Nefh-Cre;Nf2 ^fl/+^ mice, correlating with the appearance of macroscopic nerve swelling and schwannoma-like structures following nerve injury.

### Tumor development in P0-Cre;Nefh-Cre;Nf2 ^fl/+^ mice occurs without detectable LOH

**Fig. 6:**
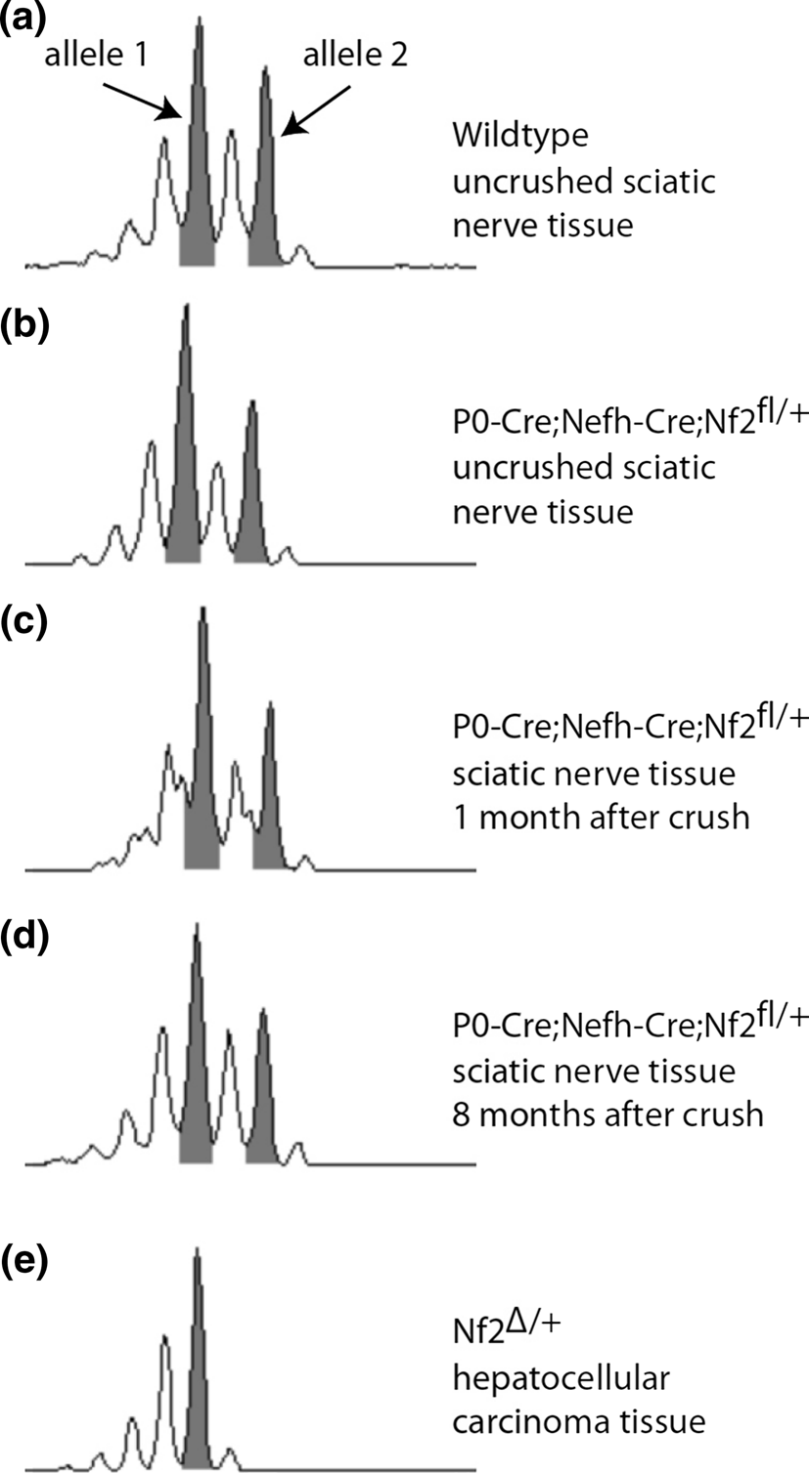
LOH of *nf2* gene is dispensable for schwannoma formation. **a**–**e** Microsatellite analysis of genomic DNA using a polymorphic marker in intron 5 of the murine *nf2* gene. Pooled DNA from at least three sciatic nerves per indicated genotype was used to detect loss of heterozygosity. Tissue from hepatocellular carcinoma of Nf2^Δ/+^ mice was used as positive control for LOH. *Grey highlighted peaks* represent the two gene alleles

Because neuropathologically validated schwannoma-like growth only occurred in crushed nerves of mice bearing deletion of one *nf2* allele in both glial and neuronal compartments of peripheral nerves (P0-Cre;Nefh-Cre;Nf2^fl/+^), we hypothesized that LOH of the *nf2* gene is dispensable for tumor initiation. Furthermore, we also aimed to determine whether LOH might occur during later stages of tumor progression. Using a polymorphic marker in intron 5 of the genomic *nf2* sequence, we could detect two alleles—illustrated by two peak groups in nerve tissue from 2-month-old wild-type animals without crush injury (Fig. 6a). Strikingly, a similar pattern was also seen for the marker amplified from genomic DNA of nerve tissue taken from 2-month-old P0-Cre;Nefh-Cre;Nf2^fl/+^ animals without crush (Fig. 6b), as well as either 1 month (Fig. 6c) or 8 months (Fig. 6d) after crush injury. In stark contrast, hepatocellular carcinoma tissue derived from mice bearing a complete heterozygous knockout of merlin, revealed a clear and total loss of the second *nf2* allele using the same method (Fig. 6e). In line with our findings, at the protein level, merlin could be detected in nerve lysates of all tested genotypes 8 months after crush injury (Supplementary Fig. 11).

In summary, relevant LOH of the *nf2* gene could not be detected in our schwannoma induction model. Considering the inherent resolution-limitation of microsatellite analysis as the standard technique for LOH detection, *nf2* LOH cannot be excluded in a minor subset of cells.

### Macrophage presence in P0-Cre;Nefh-Cre;Nf2 ^fl/+^ animals

**Fig. 7:**
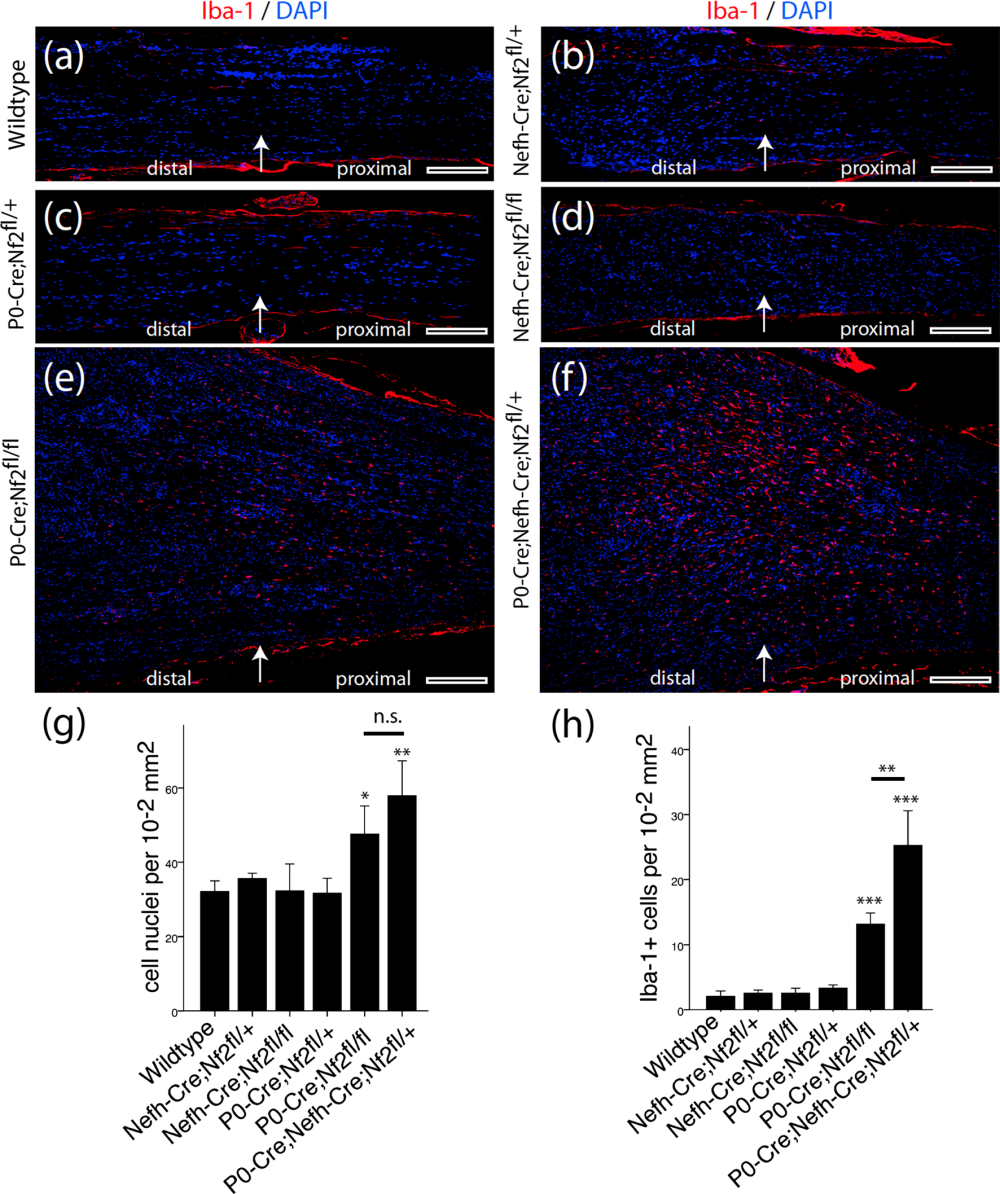
Sustained inflammatory response after nerve crush in P0-Cre;Nefh-Cre;Nf2^fl/+^ mice. **a**–**f** Longitudinal sciatic nerve sections were prepared from indicated genotypes 8 months after crush injury and immunohistochemically stained for the macrophage marker Iba-1 (*red*). DAPI counterstaining indicates cell nuclei (*blue*). *Arrow* in each image shows the position of the nerve crush. Orientation of nerves is stated as ‘distal’ and ‘proximal’. *Scale bars* represent 200 μm. **g** Quantification of cellular density in nerve tissue 8 months after crush injury, as measured by the number of DAPI-positive cell nuclei per area of tissue (one-way ANOVA analysis: ***P < 0.001; TMCT comparisons are depicted in the graph: *P < 0.05; **P < 0.01; *n.s*. not significant; *n* = 3 nerves per genotype; mean ± SD). **h** Quantification of Iba-1-positive cells in nerve tissue 8 months after crush injury (one-way ANOVA analysis: ***P < 0.001; TMCT comparisons are depicted in the graph: **P < 0.01; ***P < 0.001; *n* = 3 nerves per genotype; mean ± SD)

In addition to dissecting the molecular signaling pathways leading to schwannoma development in our mouse models, we analyzed the sciatic nerve tissue for an unresolved inflammatory response at 8 months after crush injury. In fact, in many tumor entities, cells of the innate immunity, especially macrophages, have a strong impact on tumorigenic development [1]. Furthermore, the essential role of macrophages during peripheral nerve regeneration and Wallerian degeneration is well known [34]. In sciatic nerves from P0-Cre;Nf2^fl/+^, Nefh-Cre;Nf2^fl/+^ and Nefh-Cre;Nf2^fl/fl^ animals, as well as their wild-type littermates, Iba-1 immunoreactivity indicative of macrophages was not detectable beyond unspecific background staining (Fig. 7a–d). However, in nerves taken from P0-Cre;Nf2^fl/fl^ animals (Fig. 7e) and even more prominently from P0-Cre;Nefh-Cre;Nf2^fl/+^ mice (Fig. 7f), macrophages were present in relevant numbers. Cell number quantification revealed not only a total increase of cellular density in nerve sections of these two genotypes (Fig. 7g), but also a significantly enhanced and long-lasting presence of macrophages 8 months after crush injury (Fig. 7h). Based on these quantifications, macrophages account for 19 ± 6 % (P0-Cre;Nf2^fl/fl^) and 36 ± 5 % (P0-Cre;Nefh-Cre;Nf2^fl/+^) of the total increase in cell number compared to wild-type nerve tissue. Strikingly, tumorlet-developing nerves from P0-Cre;Nefh-Cre;Nf2^fl/+^ animals contained the largest number of macrophages (Fig. 7h). Likewise, the occurrence of macrophages in schwannoma tissue could be confirmed in another established schwannoma mouse model (Supplementary Fig. 12), wherein all Postn-Cre;Nf2flox animals spontaneously develop schwannomas by 10 months of age [13].

**Fig. 8:**
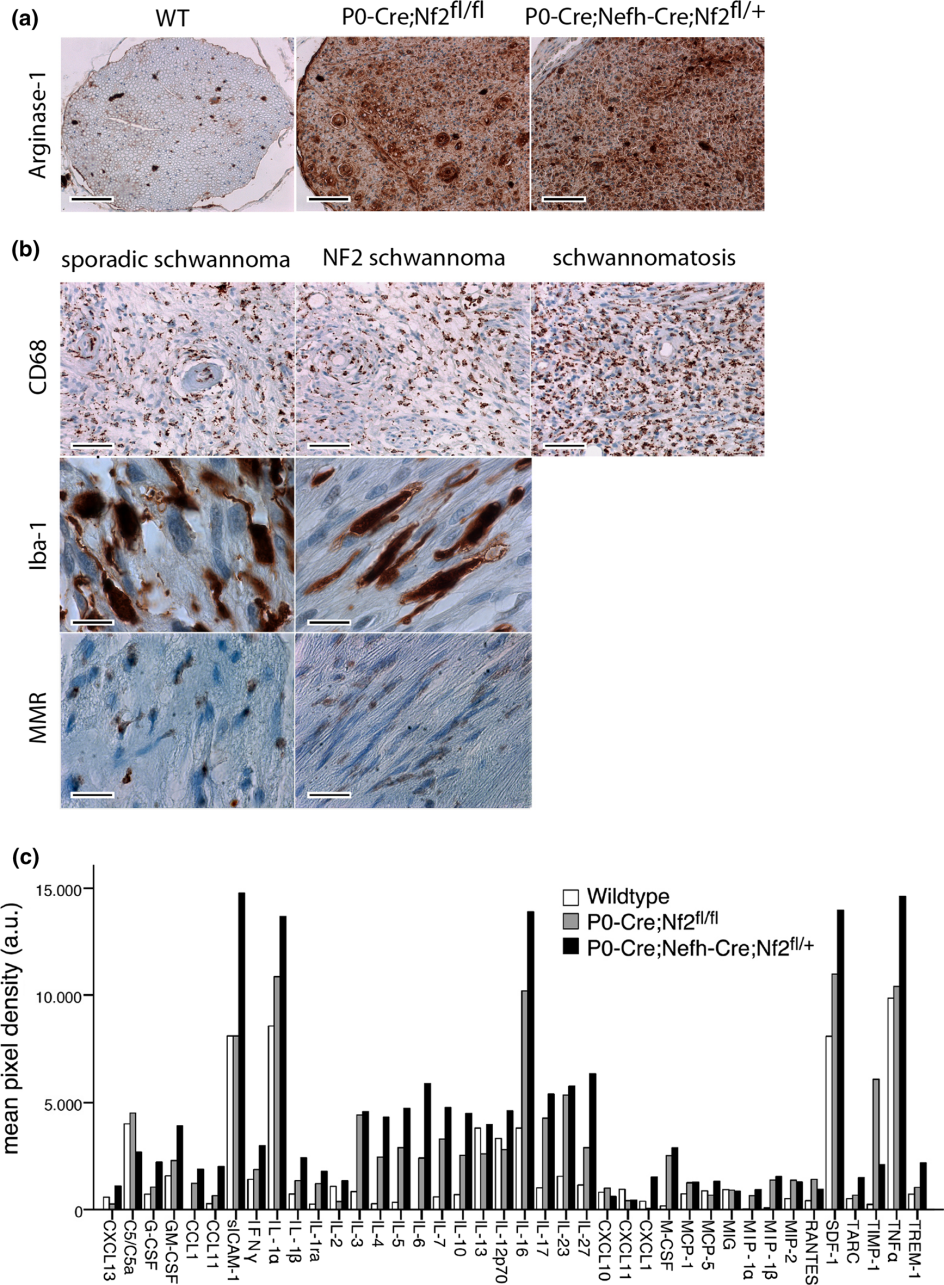
Appearance of M2-type macrophages in merlin-deficient nerves after crush injury and human schwannoma samples. **a** Representative images of sciatic nerve cross sections from indicated genotypes. Immunolabeling of Arginase-1 as a marker for M2-type macrophages shows strong expression in P0-Cre;Nf2^fl/fl^ and P0-Cre;Nefh-Cre;Nf2^fl/+^ mice, in comparison to wild-type (WT) littermates. *Scale bars* represent 100 μm. **b** Representative images of human schwannoma samples from a tissue microarray. Immunostainings against macrophage markers CD68 and Iba-1, as well as M2-type macrophage marker MMR/CD206, indicate macrophage occurence in sporadic, NF2-associated and Schwannomatosis-associated schwannomas. *Scale bars* represent 50 μm (*upper panel*), 10 μm (*middle panel*) and 20 μm (*lower panel*), respectively. **c** Cytokine levels in pooled lysates of at least four individual sciatic nerves per indicated genotype (*n* = 2). Densitometric quantification is shown as median value. For full-length blots see Supplementary Fig. 13

The relevance and significance of chronic inflammation for the development and progression of schwannomas are not fully understood. However, intratumoral inflammation in schwannomas positively correlated with tumor size and growth in a retrospective study [11]. Macrophages are functionally plastic cells with distinct polarization states. While M1-type macrophages are thought to inhibit cell proliferation, M2-type macrophages promote cell proliferation with high tissue-remodeling activity [31]. Hence, tumor-associated macrophages primarily consist of M2-type macrophage populations with little cytotoxicity towards tumor cells, because of their clearly defined cytokine armamentarium [26]. Therefore, we tested for the presence of M2-type macrophages in sciatic nerve sections of P0-Cre;Nefh-Cre;Nf2^fl/+^ mice, using an antibody raised against Arginase-1 [27]. Indeed, Arginase-1 immunoreactivity was strongly present in nerve cross sections of P0-Cre;Nf2^fl/fl^ and P0-Cre;Nefh-Cre;Nf2^fl/+^ animals compared to wild-type mice (Fig. 8a); suggesting the presence of M2-type macrophages in experimentally induced Schwann cell tumors. Importantly, M2-type macrophage immunolabeling of sciatic nerve samples showed a strong correlation with ErbB2 expression (*P* < 0.001). Ultimately, macrophage occurrence indicated by both CD68 and Iba-1 expression was also found in 28 out of 30 human biopsy samples from sporadic schwannomas, 9 out of 10 NF2-related schwannomas and in 4 out of 4 investigated Schwann cell tumors associated with schwannomatosis (Fig. 8b). Using the macrophage mannose receptor MMR/CD206 [53] as an additional non-lysosomal marker, M2-type macrophages were observed in human tissue of both sporadic and NF2-associated schwannomas (Fig. 8b).

Schwann cells have repeatedly been shown to produce chemotactic cues for macrophages, e.g., by macrophage chemoattractant protein-1, leukemia inhibitory factor and different interleukins [54]. In order to identify soluble factors that might be causative for sustained macrophage presence, with the highest intensity in nerves of P0-Cre;Nefh-Cre;Nf2^fl/+^ animals, we performed an unbiased detection of 40 different cytokines in sciatic nerve lysates. Notably, we found various cytokines to be differentially present in tissue from wild-type mice, P0-Cre;Nf2^fl/fl^ animals and schwannoma-developing P0-Cre;Nefh-Cre;Nf2^fl/+^ mice (Fig. 8c and Supplementary Fig. 13). Several cytokines with known association to chronic inflammation (such as TNFα, IL-6 and IL-10) showed the highest levels in P0-Cre;Nefh-Cre;Nf2^fl/+^ mice [23]. Furthermore, IL-6 and SDF-1/CXCL12, which exhibited the strongest levels in P0-Cre;Nefh-Cre;Nf2^fl/+^ animals, are reportedly capable of inducing transactivation of ErbB2 receptor in a ligand-independent mechanism [7, 41].

**Fig. 9:**
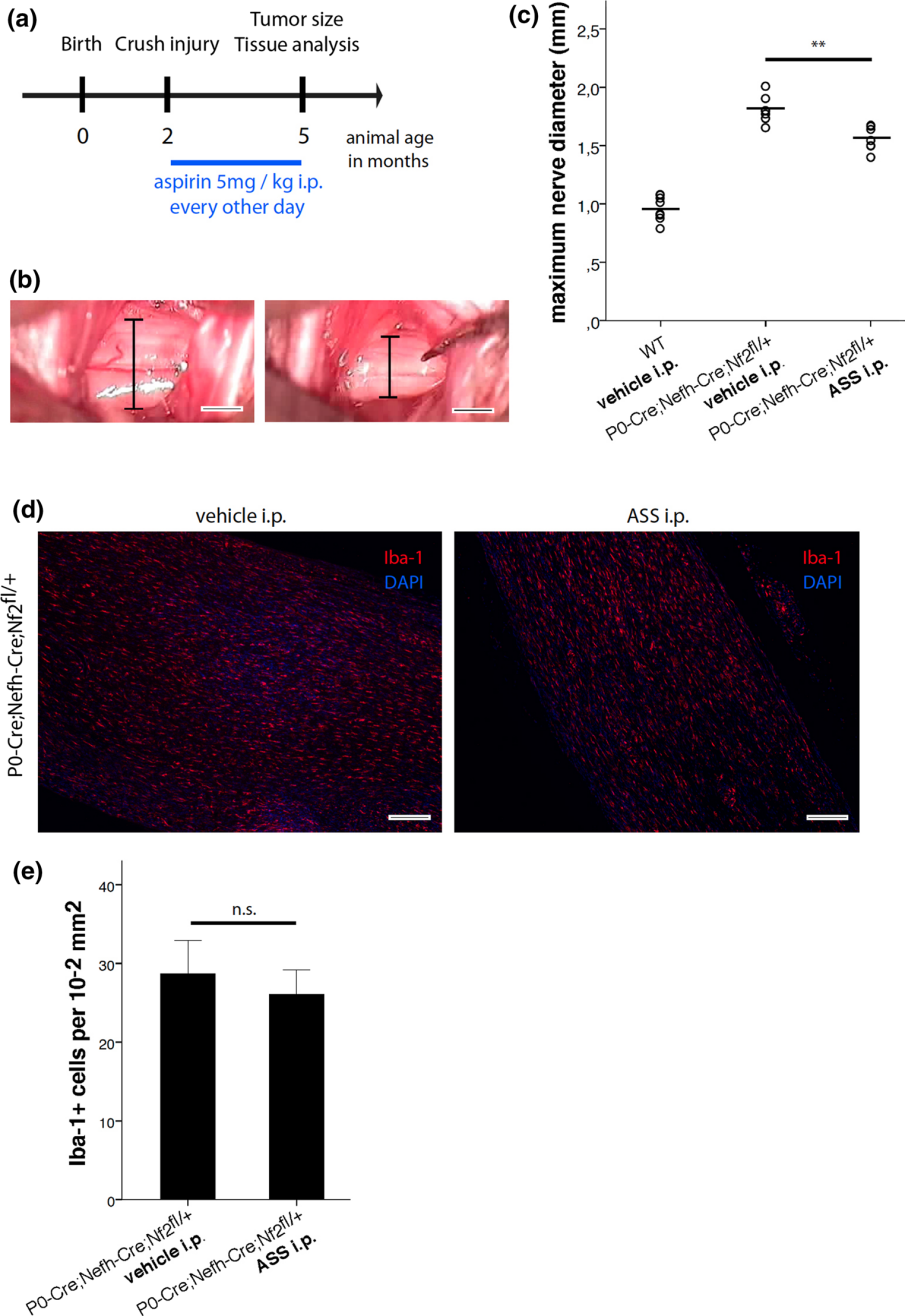
Systemic aspirin administration decreases schwannoma progression in P0-Cre;Nefh-Cre;Nf2^fl/+^ mice. **a** Schematic representation of the aspirin (ASS) treatment protocol. **b** Representative images showing the method for in situ tumor size quantification. The crushed sciatic nerve was exposed surgically in order to assess the maximum sciatic nerve diameter following as indicator for tumor size. **c** Quantification of maximum sciatic nerve diameters in wild type (WT) and P0-Cre;Nefh-Cre;Nf2^fl/+^ mice, 3 months after crush injury. Mice received systemic administration of either medium-dose aspirin (5 mg per kg ASS i.p.) or vehicle (**P < 0.01;* n* = 7 mice per genotype; mean ± SD). **d** Longitudinal sciatic nerve sections were prepared from vehicle or aspirin-treated P0-Cre;Nefh-Cre;Nf2^fl/+^ mice 3 months after crush injury and immunohistochemically stained for the macrophage marker Iba-1 (*red*). DAPI counterstaining indicates cell nuclei (*blue*). Scale bars represent 200 μm. **e** Quantification of Iba-1-positive cells nerve tissue 3 months after crush injury, taken from aspirin and vehicle-treated P0-Cre;Nefh-Cre;Nf2^fl/+^ mice (*n.s*. not significant; *n* = 3 nerves per genotype; mean ± SD)

In order to experimentally test a cause and effect relationship between chronic inflammation and schwannoma growth, we administered medium-dose aspirin (5 mg per kg equates to a dose of approximately 375 mg for humans) as commonly used anti-inflammatory drug to P0-Cre;Nefh-Cre;Nf2^fl/+^ mice after tumor induction by nerve crush injury (Fig. 9a). Following systemic treatment through intraperitoneal injections for 3 months, tumor size was quantified in situ (Fig. 9b and Supplementary Fig. 14). Strikingly, aspirin-treated P0-Cre;Nefh-Cre;Nf2^fl/+^ animals had significantly smaller tumors than vehicle-treated mice of the same genotype, as indicated by a relevant reduction in the maximum sciatic nerve diameter. Subsequent immunohistochemical analysis of longitudinal nerve sections indicated a decrease in the absolute number of macrophages (Fig. 9d), but not when related to the area of tissue section (Fig. 9e).

Taken together, our data highlights an unresolved inflammation in two different mouse models of schwannoma development and human schwannoma tissue. For the first time, we provide causal evidence for the relevance of chronic inflammation in schwannoma development, as anti-inflammatory treatment significantly reduces tumor progression in our in vivo disease model.

## Discussion

Schwann cell-intrinsic signaling mechanisms leading to tumorigenic transformation and schwannoma development have been explored in great detail. However, the identification of diversified signaling events related to the merlin tumor suppressor protein has yet to lead to the development of better therapeutic options for the management of sporadic or NF2-related schwannomas. Therefore, beyond ‘watchful waiting’, surgical resection of nerve sheath tumors is often the only available treatment. This study aimed to investigate the importance of the nerve microenvironment and Schwann cell-extrinsic factors for the development of schwannomas. In line with previous findings suggesting a key role for axons in Schwann cell control [9], we provide experimental evidence that the axonal compartment of peripheral nerves contributes to schwannoma formation. In fact, 12 of the 13 animals with the heterozygous knockout of the *nf2* gene in neurons and Schwann cells (P0-Cre;Nefh-Cre;Nf2^fl/+^), developed macroscopic nerve swellings at the same position where a single crush injury had been performed 8 months earlier. Strikingly, the marked swelling was not just restricted to the crush site, but expanded several millimeters into the distal part of the nerve where branching of the sciatic nerve into tibial and common peroneal nerve occurs. Crush injury-induced macroscopic nerve swelling was also observed in another mouse model frequently used to mimic NF2 disease by conditional biallelic *nf2* knockout in Schwann cells (P0-Cre;Nf2^fl/fl^). However, neuropathological assessment of the macroscopic nerve swellings revealed that NF2 patient-resembling tumors and Schwann cell de-differentiation only occurred in animals with cooperative *nf2* gene loss in axons and adjacent Schwann cells (P0-Cre;Nefh-Cre;Nf2^fl/+^). Our findings suggest that axon-derived instructive cues for Schwann cells might be implicated in schwannoma formation. Because of the bidirectional communication between cells and their surrounding context, the microenvironment has been proven to be critical for both tissue homeostasis and tumor growth [42]. Consistent with previous work from our group and others, we provide more evidence that the Neuregulin1-ErbB network, crucial for normal cell-to-cell communication between Schwann cells and axons, is persistently disturbed in merlin-deficient nerve tissue.

The reason for the preference of the vestibular nerve for schwannoma growth remains profoundly obscure [52]. However, our data raise the question of whether mechanical irritation of nerves or local nerve injury, respectively, can act as predilection sites for schwannoma development—comparable to neurofibroma, which are often viewed as unrepaired wounds [3, 43]. Remarkably, NF2-related schwannomas often occur at localizations where peripheral nerves are in close proximity to bony structures, e.g., spinal nerves emerging from the spinal column through the intervertebral foramen and the vestibular nerve, which uses the internal auditory meatus as a passageway. However, while the connection between neuromas and de-regulated nerve regeneration after injury (traumatic neuroma) is widely accepted [6], schwannoma formation after mechanical nerve irritation has not been studied in a systematic manner.

Wounding of the sciatic nerve has previously been shown to induce a transient inflammatory response involving macrophages [34]. On the other hand, sustained or chronic tissue inflammation is known to drive tumor formation [25]. In our model, we can clearly correlate the formation of schwannomas with the presence of macrophages. Furthermore, we provide evidence that at least a subset of macrophages shows ‘alternatively activated’ M2 polarization, which can exert anti-inflammatory but pro-tumorigenic functions. Importantly, in each mouse line of this study, macrophages have a wild-type genetic setup and are spared from genetic modifications. The finding that virtually all sporadic and NF2-associated human schwannomas tested in this study show macrophage presence, further supports the concept that schwannoma formation is promoted by an unresolved inflammatory response. In line with our own causal evidence, a recent retrospective study found that aspirin intake as an anti-inflammatory drug correlated with slowed growth of vestibular schwannomas in patients [20].

The results presented here enable us to clearly correlate the appearance and number of non-differentiated, non-myelinating, Schwann cells at the lesion site with the occurrence of macrophages. This in turn coincides with substantial cellular proliferation resulting in tumorlet formation, identical to NF2-related schwannomas again underlining the notion that tumors do not diversify solely in terms of genetic alterations, but also with respect to the nature of their microenvironment [42].

The necessity of bi-allelic *Nf2* gene (e.g., due to LOH) for schwannoma development for human individuals is a long-standing clinical hypothesis. However, a growing number of tumor suppressor genes have been shown not to conform to the classical two-hit hypothesis proposed by Alfred Knudson [37]. In fact, the data on LOH frequency in schwannoma samples are not entirely conclusive throughout the existing literature. Therefore, we aimed to investigate whether LOH might not be a necessary prerequisite for schwannoma initiation. From the mouse model and the LOH detection technique applied in this study, we conclude that LOH of the *nf2* gene is neither required for initiation nor progression of schwannomas, considering the inherent detection limit of the chosen technique. Nonetheless, other genetic mutations might occur during the course of schwannoma growth that remain to be detected or considered. This potential necessity for other genetic events to occur to prompt schwannoma formation is further supported by our results from P0-Cre;Nf2^fl/fl^ animals—where nerve injury is not sufficient to induce schwannomas, despite biallelic *nf2* gene inactivation. We therefore suggest performing a systematic and unbiased analysis of genetic alterations found in human schwannoma samples, e.g., by whole-genome sequencing.

In essence, our data suggest that a Schwann cell-focused pathogenesis of schwannoma development is insufficient. Instead, we provide evidence for a multi-leveled importance of the nerve microenvironment, where instructive cues from axons and macrophage presence play important roles in combination with failed regenerative processes of afflicted nerves. Clearly, microenvironmental considerations further complicate the already challenging task of understanding tumors. On the other hand, this increasingly complex construct of tumor development presents more opportunities for the identification of potential new therapeutic targets, urgently needed for debilitating tumor predisposition syndromes like NF2 disease.

## Electronic supplementary material

Below is the link to the electronic supplementary material.

(1) Table showing all genetically engineered mouse lines used in this study. (2) Representative semi-thin cross sections of the distal part of intact non-crushed sciatic nerves of 10-month-old mice. Genotypes as indicated. Scale bars represent 20 μm. (3) Axon diameter quantification of intact non-crushed sciatic nerves of 10-month-old mice of indicated genotypes (**P < 0.01; ***P < 0.001; n = 3 nerves per genotype; n > 60 axons per genotype; mean ± SD). (4) Quantification of mean solidity factor as a parameter for roundness of axons was performed on intact non-crushed sciatic nerves of 10-month-old mice of indicated genotypes (*P < 0.05; **P < 0.01; ***P < 0.001; n = 3 nerves per genotype; n > 55 axons per genotype; mean ± SD). (5) Quantification of mean myelin thickness of axons was performed on intact non-crushed sciatic nerves of 10-month-old mice of indicated genotypes (*P < 0.05; n = 3 nerves per genotype; n > 60 axons per genotype; mean ± SD). (6) Quantification of the g-ratio of intact non-crushed sciatic nerves of 10-month-old mice of indicated genotypes (*P < 0.05; **P < 0.01; ***P < 0.001; n = 3 nerves per genotype; n > 55 axons per genotype; mean ± SD). G-ratio represents the ratio of the inner axonal diameter to the total outer diameter of the nerve fiber. (7) Representative image of HE-stained sciatic nerve cross sections from P0-Cre;Nefh-Cre;Nf2^fl/+^ mice, 8 months after crush injury. Arrow indicates disorganized tumorlet-like structure as shown in Fig. 2f. Scale bar represents 50 μm. (8) Immunohistochemical stainings of longitudinal sciatic nerve sections prepared from indicated genotypes, 8 months after crush injury. Immunolabeling of p75 and myelin basic protein (MBP) indicates immature/non-myelinating Schwann cells and myelination, respectively. Arrow in each image shows the position of the nerve crush. Orientation of nerves is stated as ‘distal’ and ‘proximal’. Scale bars represent 100 μm. (9) Full-length blot of Fig. 5a (pooled tissue from at least three different animals per indicated genotype was prepared from crushed and intact sciatic nerves). Immunoblot for receptor tyrosine kinase ErbB2, Neuregulin 1 type III (Nrg1 type III), phospho-c-Jun and GAPDH as loading control (n = 3). (10) Full-length blot of Fig. 5b (pooled tissue from at least three different animals per indicated genotype was prepared from crushed and intact sciatic nerves). Immunoblot for Erk1/2 and phospho-Erk1/2 (pErk1/2). Densitometric quantification of pErk1/2: Erk1/2 ratio was normalized to uncrushed nerve tissue for each genotype (n = 3). (11) Immunoblot of sciatic nerve lysates for merlin and GAPDH as loading control (n = 3). Pooled tissue from at least three different animals per indicated genotype was prepared from crushed and intact sciatic nerves. (12) DRG schwannoma sections of two individual 10-month-old Postn-Cre;Nf2flox mice were immunohistochemically stained for the macrophage marker Iba-1 (staining in brown). Scale bars represent 20 μm. (13) Dot blots for cytokine detection used for the quantification of cytokine levels in Fig. 8c. Assay overlay is shown as provided by the manufacturer. Pooled lysates from at least three sciatic nerves per indicated genotype were used. (14) Representative, dissected sciatic nerves from 5-month-old P0-Cre;Nefh-Cre;Nf2^fl/+^ mice receiving either systemic aspirin treatment or vehicle control for 3 months. Crushed nerves (3 months post-injury) are shown at the top; intact nerves are depicted at the bottom of each representative image. Proximal nerve parts are on the right side of the images; distal parts on the left side. Scale bars represent 2 mm (PDF 29658 kb)
