# Supplementary material for: The importance of nerve microenvironment for schwannoma development
Source: Acta Neuropathol. 2016 May 28;132:289–307. doi: 10.1007/s00401-016-1583-8 (PMC4947119; doi:10.1007/s00401-016-1583-8)

1 Mouse lines used in this study

Mouse line	Description
WT	Wildtype animals
P0-Cre;Nf2$^{fl/+}$	Heterozygous loss of merlin in Schwann cells
P0-Cre;Nf2$^{fl/fl}$	Homozygous loss of merlin in Schwann cells
Nefh-Cre;Nf2$^{fl/+}$	Heterozygous loss of merlin in neurons
Nefh-Cre;Nf2$^{fl/fl}$	Homozygous loss of merlin in neurons
P0-Cre;Nefh-Cre;Nf2$^{fl/+}$	Heterozygous loss of merlin in Schwann cells and neurons

2

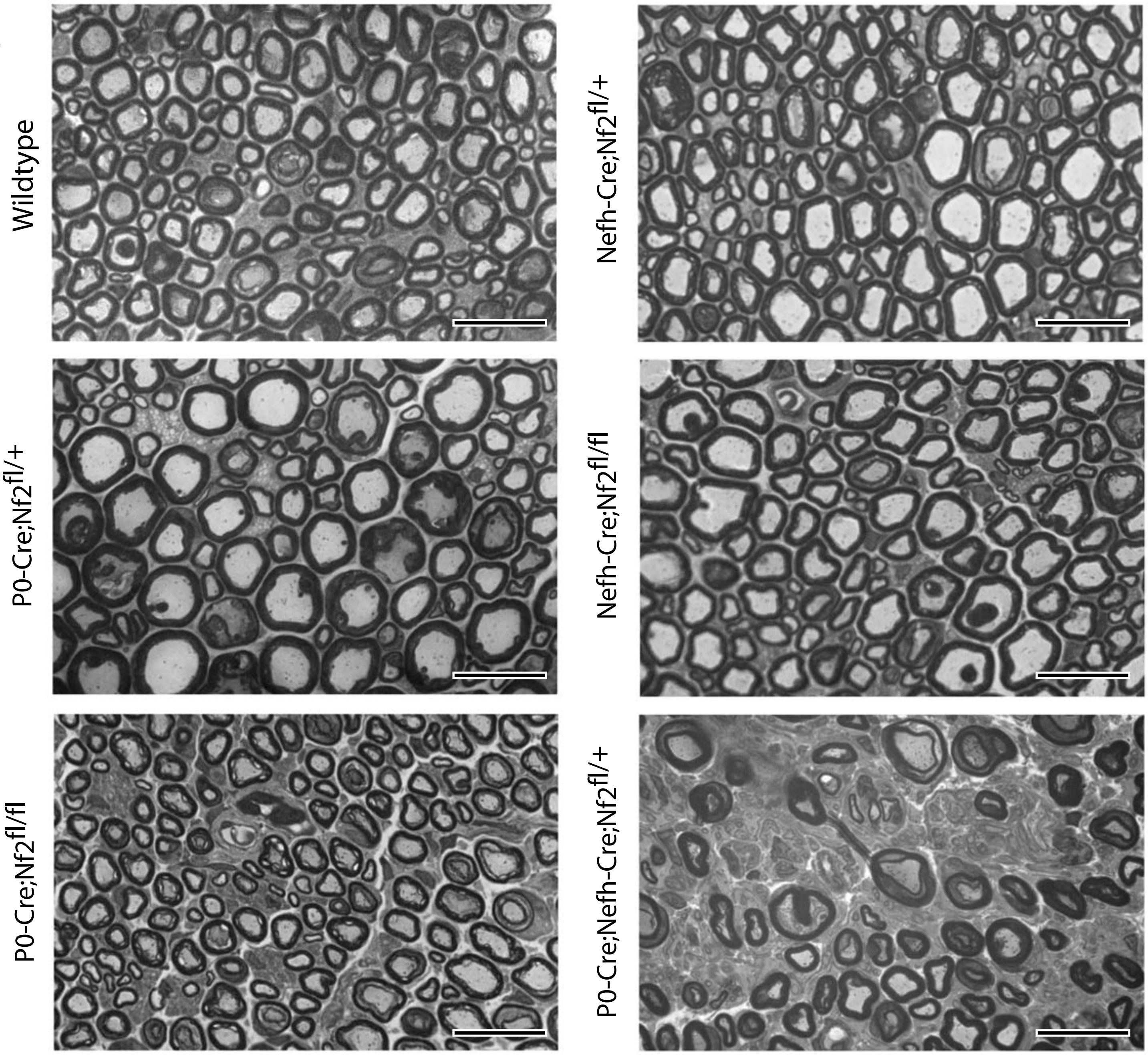

3

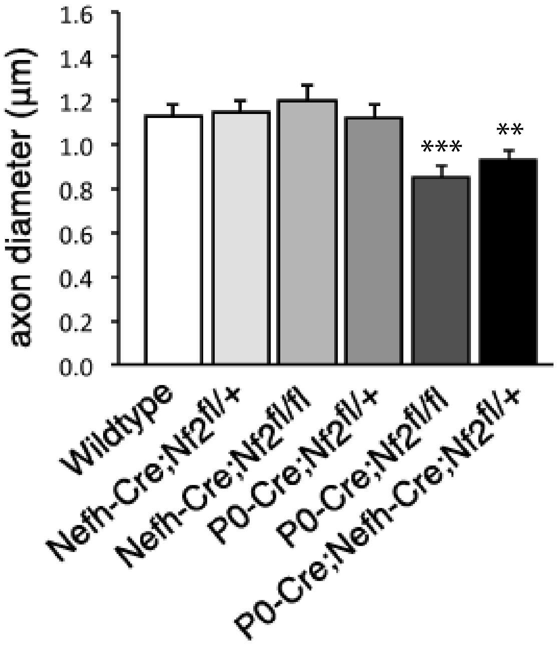

4

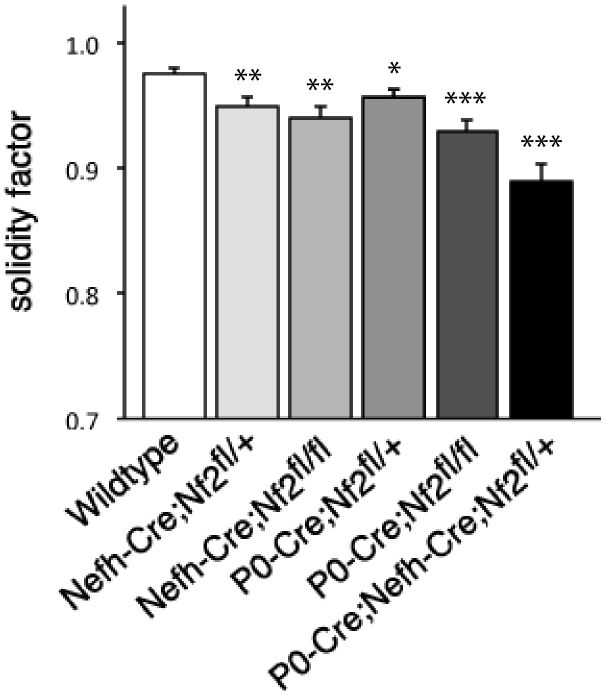

5

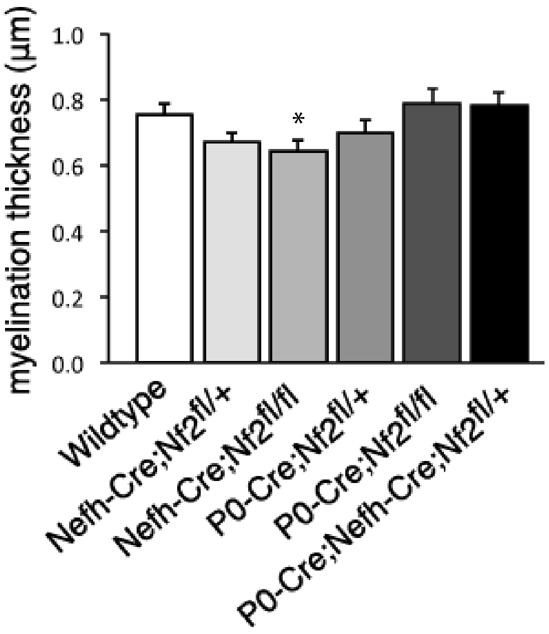

6

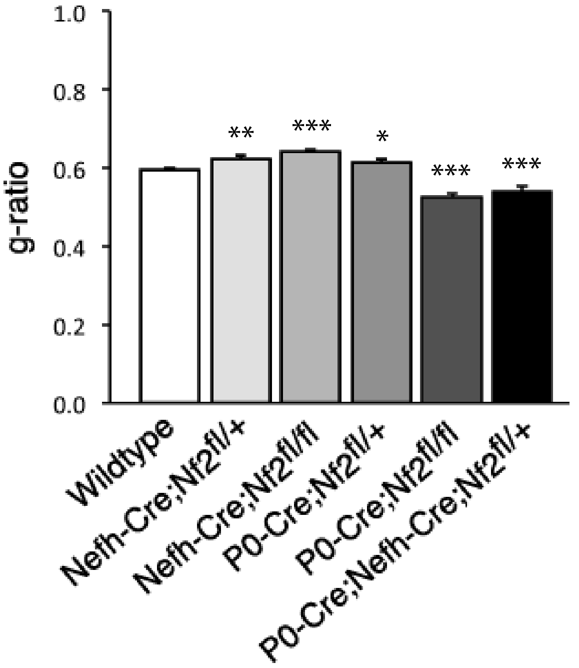

HE staining

7

P0-Cre;Nefh-Cre;Nf2$^{fl/+}$

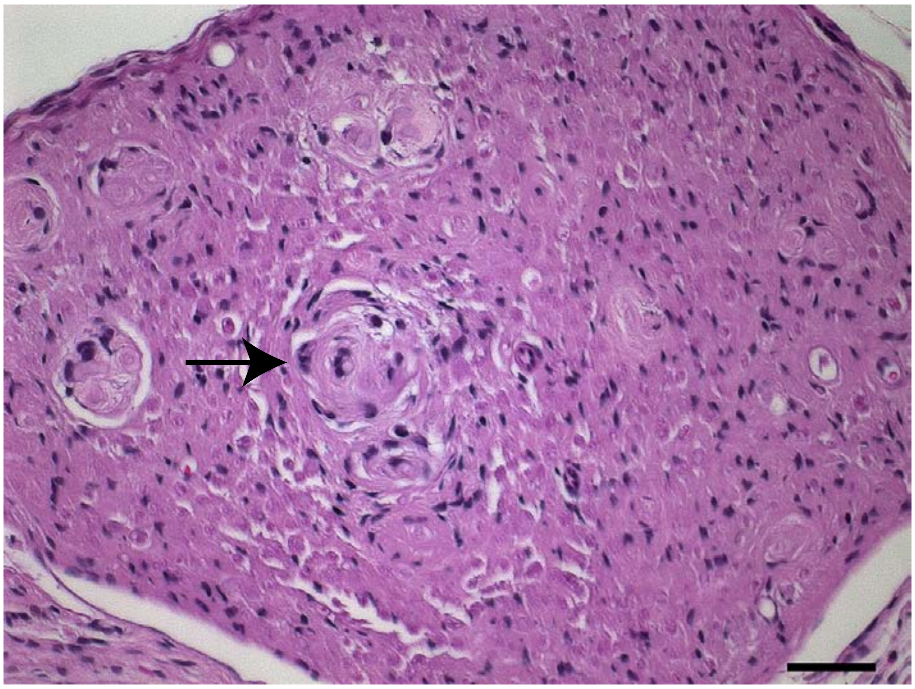

Supplementary Figures

8

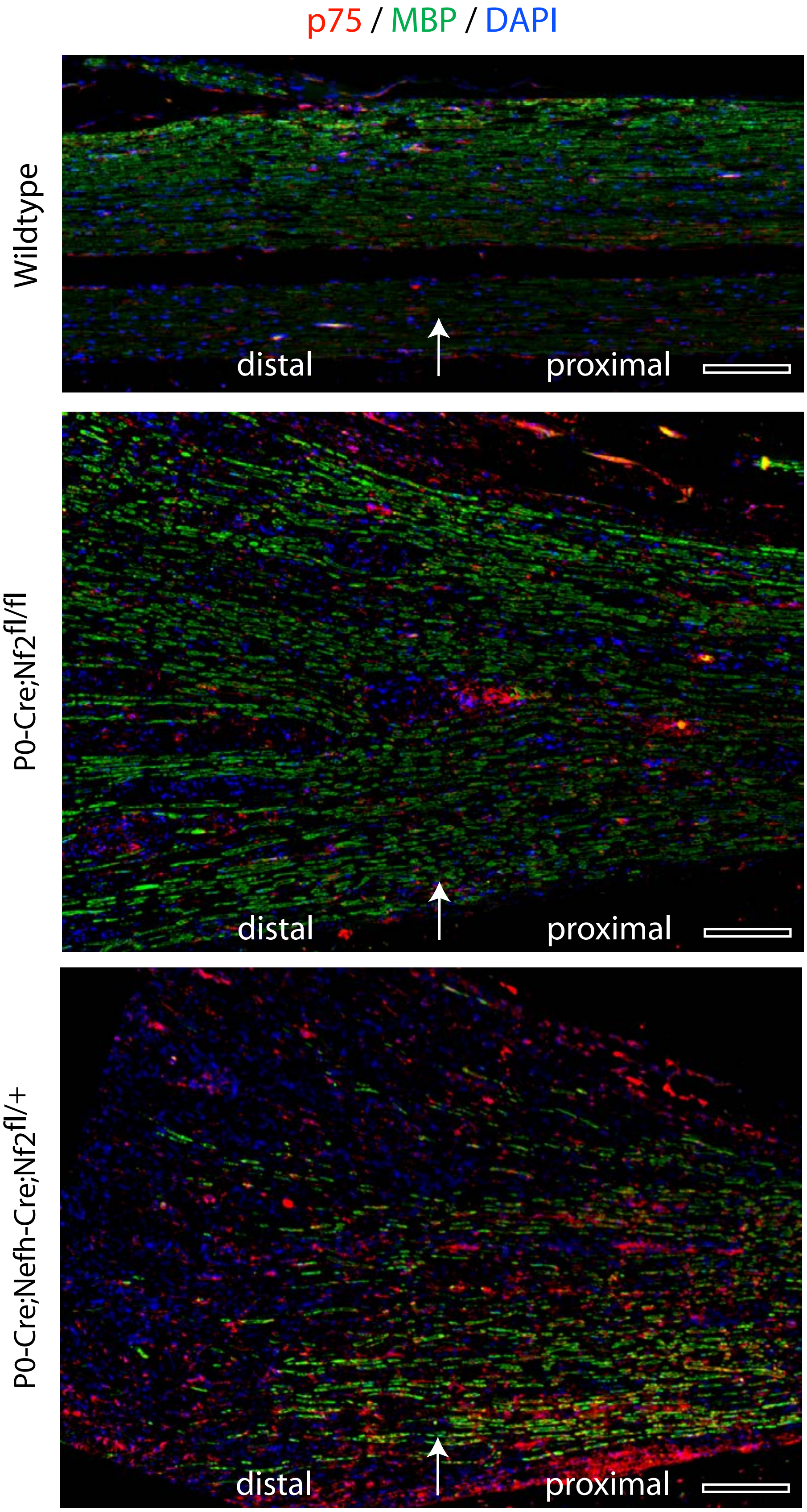
p75 / MBP / DAPI
Wildtype
P0-Cre;Nf2fl/fl
P0-Cre;Nefh-Cre;Nf2fl/+
distal
proximal
distal
proximal
distal
proximal

9

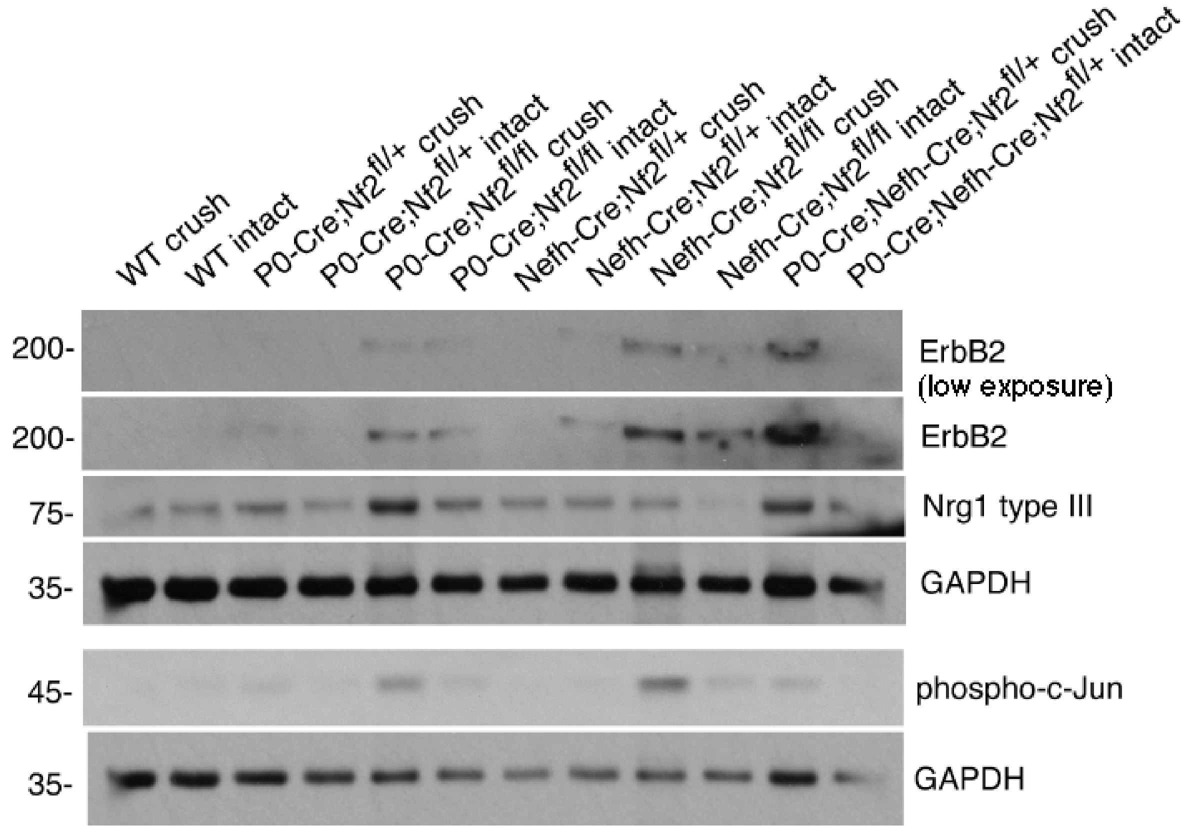
WT crush
WT intact
P0-Cre;Nf2$^{fl/+}$ crush
P0-Cre;Nf2$^{fl/+}$ intact
P0-Cre;Nf2$^{fl/fl}$ crush
P0-Cre;Nf2$^{fl/fl}$ intact
Nefh-Cre;Nf2$^{fl/+}$ crush
Nefh-Cre;Nf2$^{fl/+}$ intact
Nefh-Cre;Nf2$^{fl/fl}$ crush
Nefh-Cre;Nf2$^{fl/fl}$ intact
P0-Cre;Nefh-Cre;Nf2$^{fl/+}$ crush
P0-Cre;Nefh-Cre;Nf2$^{fl/+}$ intact
200-
ErbB2 (low exposure)
200-
ErbB2
75-
Nrg1 type III
35-
GAPDH
45-
phospho-c-Jun
35-
GAPDH

10

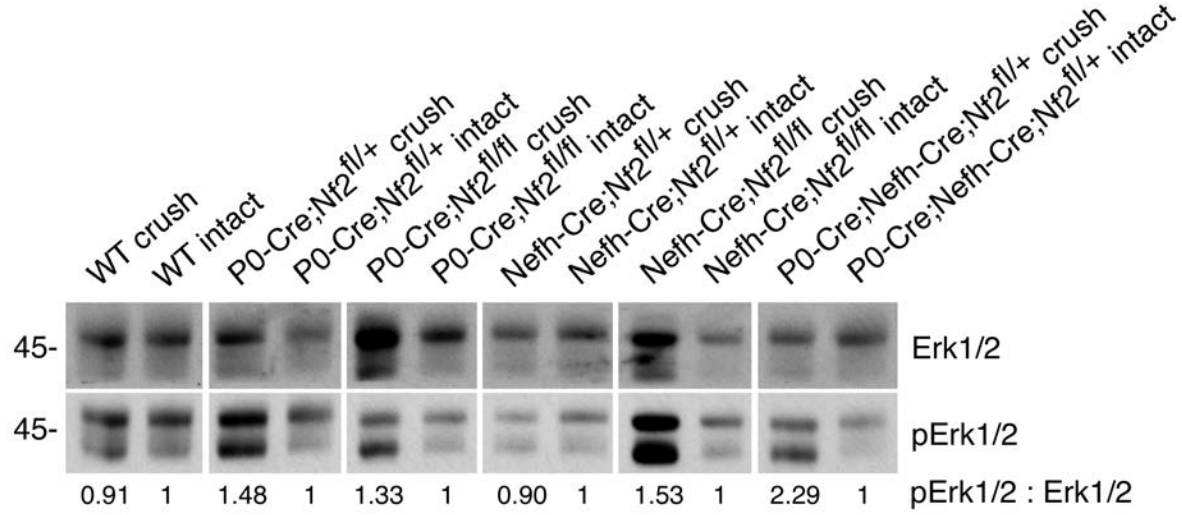
WT crush
WT intact
P0-Cre;Nf2$^{fl/+}$ crush
P0-Cre;Nf2$^{fl/+}$ intact
P0-Cre;Nf2$^{fl/fl}$ crush
P0-Cre;Nf2$^{fl/fl}$ intact
Nefh-Cre;Nf2$^{fl/+}$ crush
Nefh-Cre;Nf2$^{fl/+}$ intact
Nefh-Cre;Nf2$^{fl/fl}$ crush
Nefh-Cre;Nf2$^{fl/fl}$ intact
P0-Cre;Nefh-Cre;Nf2$^{fl/+}$ crush
P0-Cre;Nefh-Cre;Nf2$^{fl/+}$ intact
45-
Erk1/2
45-
pErk1/2
0.91 1 1.48 1 1.33 1 0.90 1 1.53 1 2.29 1
pErk1/2 : Erk1/2

11

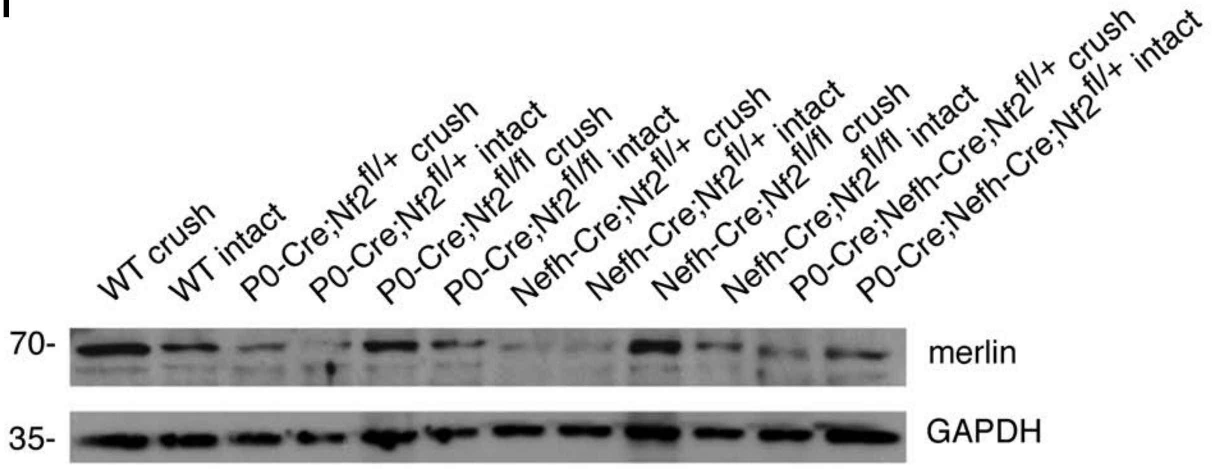

12

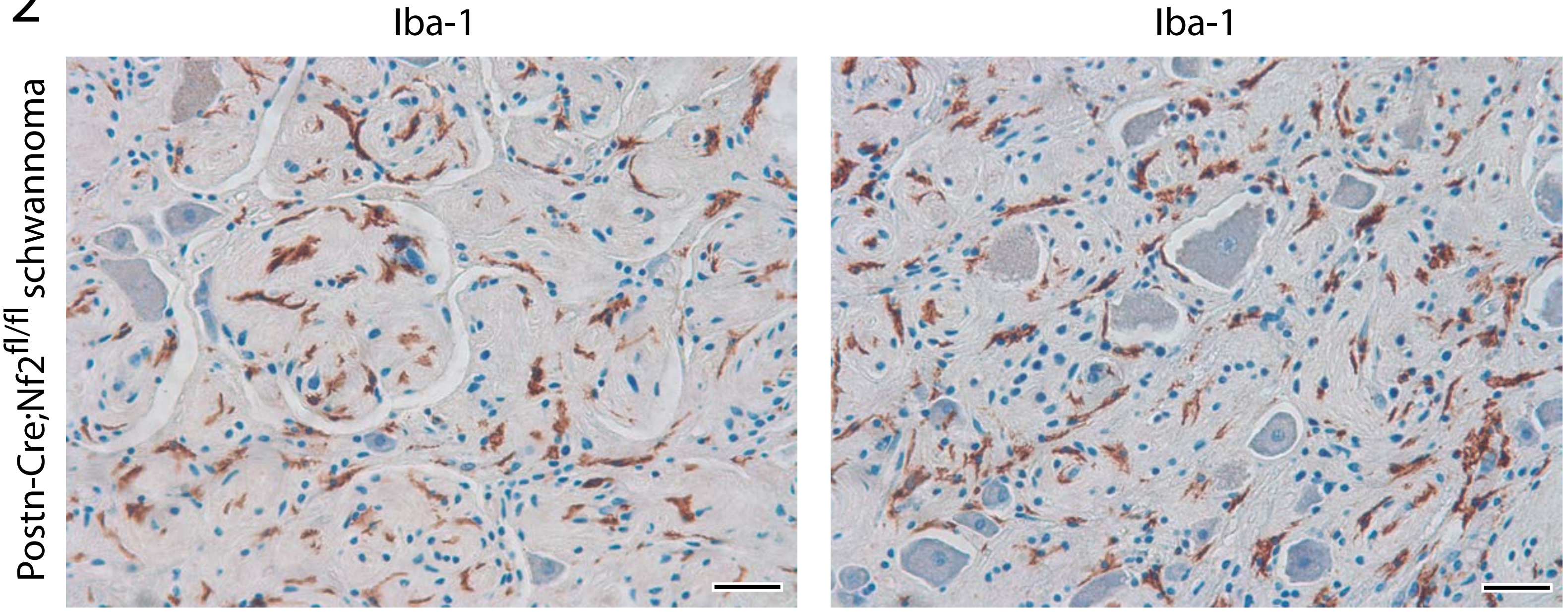

13

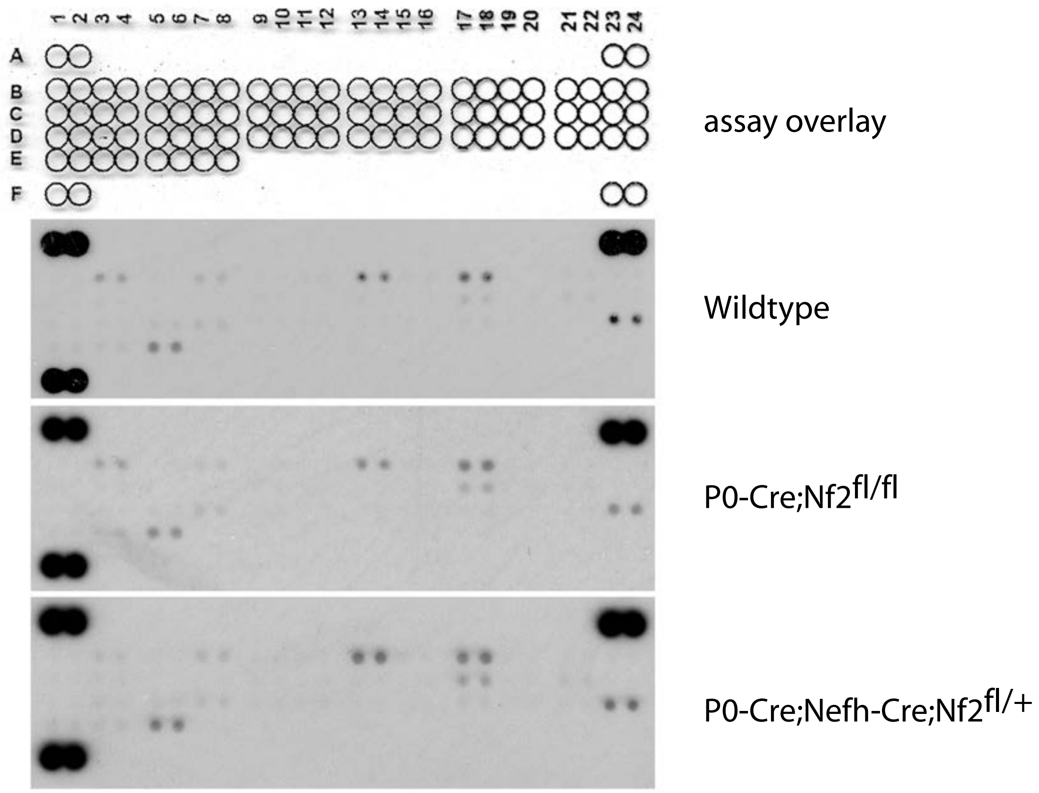

Supplementary Figures

14

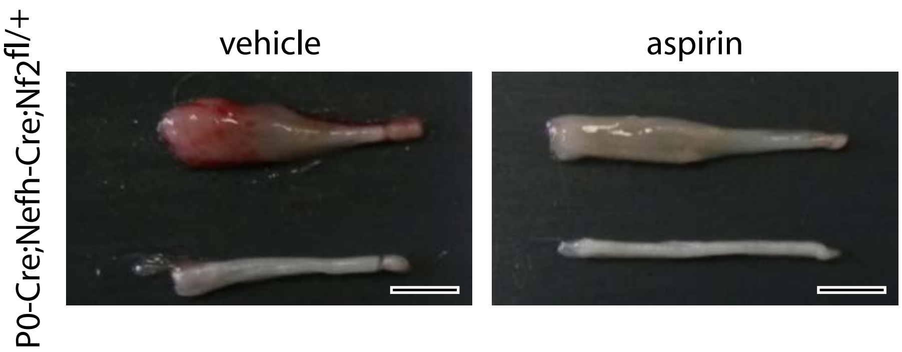

Supplement: Supplementary file 1 — (1) Table showing all genetically engineered mouse lines used in this study. (2) Representative semi-thin cross sections of the distal part of intact non-crushed sciatic nerves of 10-month-old mice. Genotypes as indicated. Scale bars represent 20 μm. (3) Axon diameter quantification of intact non-crushed sciatic nerves of 10-month-old mice of indicated genotypes (**P < 0.01; ***P < 0.001; n = 3 nerves per genotype; n > 60 axons per genotype; mean ± SD). (4) Quantification of mean solidity factor as a parameter for roundness of axons was performed on intact non-crushed sciatic nerves of 10-month-old mice of indicated genotypes (*P < 0.05; **P < 0.01; ***P < 0.001; n = 3 nerves per genotype; n > 55 axons per genotype; mean ± SD). (5) Quantification of mean myelin thickness of axons was performed on intact non-crushed sciatic nerves of 10-month-old mice of indicated genotypes (*P < 0.05; n = 3 nerves per genotype; n > 60 axons per genotype; mean ± SD). (6) Quantification of the g-ratio of intact non-crushed sciatic nerves of 10-month-old mice of indicated genotypes (*P < 0.05; **P < 0.01; ***P < 0.001; n = 3 nerves per genotype; n > 55 axons per genotype; mean ± SD). G-ratio represents the ratio of the inner axonal diameter to the total outer diameter of the nerve fiber. (7) Representative image of HE-stained sciatic nerve cross sections from P0-Cre;Nefh-Cre;Nf2fl/+ mice, 8 months after crush injury. Arrow indicates disorganized tumorlet-like structure as shown in Fig. 2f. Scale bar represents 50 μm. (8) Immunohistochemical stainings of longitudinal sciatic nerve sections prepared from indicated genotypes, 8 months after crush injury. Immunolabeling of p75 and myelin basic protein (MBP) indicates immature/non-myelinating Schwann cells and myelination, respectively. Arrow in each image shows the position of the nerve crush. Orientation of nerves is stated as ‘distal’ and ‘proximal’. Scale bars represent 100 μm. (9) Full-length blot of Fig. 5a (pooled tissue from at leas [file 401_2016_1583_MOESM1_ESM.pdf]
